# Supplementary material for: How Do Aromatic Nitro Compounds React with Nucleophiles? Theoretical Description Using Aromaticity, Nucleophilicity and Electrophilicity Indices
Source: Molecules. 2020 Oct 20;25(20):4819. doi: 10.3390/molecules25204819 (PMC7587944; doi:10.3390/molecules25204819)
Supplement: Supplementary file 1 [file molecules-25-04819-s001.pdf]

Supporting Information

Table of Contents

Geometry preparation details.	**S1**
Nucleophilicity and electrophilicity indexes calculation details.	**S2**
HOMA aromaticity index calculations details.	**S3**
Kinetic isotope effect (KIE) calculations details	**S4**
The circumference of the nitrobenzene rings along the reaction path.	**S5**
The interatomic distances changes between substrates and σ^X-adducts structures.	**S6**
Transition state geometries	**S7**
Bibliography	**S8**

S1. Geometry preparation details.

In the main text of this work we present the theoretical results obtained using Gaussian 09 package[1]. The geometries were created using GaussView 5.0. The optimization of equilibrium and transition state structures has been performed using PBE1PBE hybrid functional[2] with 6-31+G(d) basis set and the energy in each single point has been recalculated using the much more accurate basis set 6-311+G(2d,p). No geometry restrictions have been applied. The thermal and ZPE correction has been used in the calculations. Frequency analysis has been performed to find the proper ground state (no imaginary frequencies) and transition state (one imaginary frequency) structures. In the case when more than one transition state or stationary structure has been found the statistical contribution of energy and electron density (Hirschfeld electron density population, shown on Fig. 4 in the main text) has been calculated using standard Boltzmann equation. For each type of aromatic ring, the transition state structure with the lowest energy has been taken for further IRC calculations that were performed to present the reaction profiles showing the smooth connection between the substrates and intermediates or product structures. The HOMA indices[3] and Fukui functions[4] computed based on Hirschfeld population analysis were performed on selected points along the IRC routes. The computational strategy was adopted based on work published by R. Ormazábal-Toledo et al.[5,6]

The gas-phase calculation has been performed to consider electron effects that control the reaction directions. No solvation model has been used in the calculations to show pure electronic interactions in the molecular systems. The synthetic experiments are performed in nonpolar solvents[7], which is why the reproduction of solvent effects in the presented case are less important. The solute-solvent interactions, especially hydrogen bond formation, is minimized and does not affect the overall shape of the potential energy surface.[8]

The electron density population for each step of reaction paths has been calculated using Hirschfeld[9] method

.

S2. Nucleophilicity and electrophilicity indexes calculation details.

The global nucleophilicity index has been calculated using equation proposed by Jaramillo et al.[10]:

$$\omega_{global}^{-} = \frac{[\eta_{Nu^-} \times (\mu_{Nu^-} - \mu_{PG})^2]}{[2 \times (\eta_{Nu^-} + \eta_{PG})^2]}$$

The global electrophilic index has been described and argued by Parr et al.[11] as below:

$$\omega_{global}^{+} = \frac{\mu^2}{2\eta}$$

The chemical potential[12] μ is defined as

$$\mu = -\frac{1}{2}(IE - EA)$$

and chemical hardness[13] η

$$\eta = IE - EA$$

,where IE is the ionization energy defined as energetic change for the electron removal reaction. That means the IE is a energy needed to move an electron from the molecular system to infinity, namely:

$$IE = \Delta G_{N-1} - \Delta G_N$$

The second part of the equation determining the chemical hardness parameter is the electron affinity EA, which is a negative value of the energy needed to attach an electron from environment to the molecular system. Which is defined as:

$$EA = \Delta G_N - \Delta G_{N+1}$$

,where, ΔG_N is the Gibbs energy of N-electron molecular system, ΔG_{N+1} is the Gibbs energy of molecular system with additional single electron charge and ΔG_{N-1} is the Gibbs energy with single electron-deficient system. For the calculations of the global electrophilicity and nucleophilicity of the systems, ΔG_{global} has been calculated using equation:

$$\Delta G_{global} = \Delta G_{NB} + \Delta G_{Nu^-}$$

,where ΔG_{global} is a sum of Gibbs energies of nitrobenzene ΔG_{NB} and nucleophile ΔG_{Nu^-}.

Local indices of nucleophilicity and electrophilicity have been calculated using equations[5,6]:

$$\omega_{local}^{-} = \sum_{k\epsilon G} f_k^{-} \omega_{global}^{-}$$

$$\omega_{local}^{+} = \sum_{k\epsilon G} f_k^{+} \omega_{global}^{+}$$

,where k is an atom belonging to the group of atoms G andf_k^-, f_k^+ are the Fukui functions[4], described as:

$$f^-(\vec{r}) = \rho_N(\vec{r}) - \rho_{N-1}(\vec{r})$$

$$f^+(\vec{r}) = \rho_{N+1}(\vec{r}) - \rho_N(\vec{r})$$

,where $\rho_N(\vec{r})$, $\rho_{N-1}(\vec{r})\rho_{N+1}(\vec{r})$ are an electron densities, respectively for N electrons, N-1 electrons and N+1 electrons species.

S3. HOMA, aromaticity index calculations details.

The Harmonic Oscillator Model of Aromaticity (HOMA)[3] has been chosen as a descriptor of electron reorganization in aromatic ring of nitrobenzenes during the reaction path. Aromaticity index HOMA has been calculated for each step on the reaction potential energy surface using the equation mentioned below. HOMA index is based on geometrical parameters of aromatic ring coming from optimized structures on PBE1PBE/6-31+G(d) level of theory.

$$HOMA = 1 - \frac{\alpha}{n}\sum(R_{opt} - R_i)^2$$

,where *n* is a number of bonds in aromatic ring taken into the summation, α is an empirical constant, R_{opt} is the optimal value of aromatic C-C bond and R_i is the calculated bond length.

S4. Kinetic Isotope Effect (KIE) calculations details.

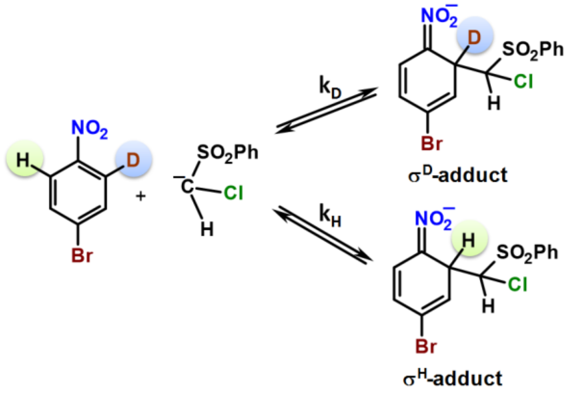

S4a. Model reactions between isotope labeled 2-deuter,4-bromonitrobenznen and the carbanion of chloromethyl phenyl sulfone ($PhSO_2CHCl^-$) as a nucleophile.

The full energy profiles of those reactions have been calculated using tree levels of theory PBE1PBE/6-311+G(2d,p)||PBE1PBE/6-31+g(d), B3LYP/6-311+G(d,p), M06-2X/6-311+G(d,p) to determine the activation energy levels. Based on those data the reaction rates and kinetic isotope effect parameter have been calculated using extension of the standard Eyring-Polanyi[14,15] equation,

$$\frac{k_H}{k_D} = e^{-\frac{\Delta\Delta G^{\neq}}{RT}}.$$

, where k is a reaction rate, T is a room temperature, $\Delta\Delta G^{\neq}$ difference between the activation energies both competitive reactions, R is universal gas constant.

S4b. The calculated free Gibbs energies ΔG [kcal/mol] for model reactions.

Method	Sub.	TS-H	σ^H add.	TS-D	σ^D add.	$\Delta\Delta G^{\neq}$	$\Delta\Delta G_{adduct}$	k_H/k_D	Exp. k_H/k_D
①	0	5.63	-4.17	5.42	-4.48	0.21	0.31	0.70	
②	0	9.17	1.38	8.89	1.63	0.28	0.25	0.62	0.8 [16]
③	0	2.02	-8.69	1.90	-8.96	0.12	0.27	0.82	

① PBE1PBE/6-311+G(2d,p)||PBE1PBE/6-31+g(d), ② B3LYP/6-311+G(d,p),③ M06-2X/6-311+G(d,p),
Sub. – substrates; σ^H, σ^D add. – adduct; $\Delta\Delta G^{\neq}$ – transition state energy difference TS-H – TS-D; $\Delta\Delta G_{adduct}$ – energy diference between σ^H adduct – σ^D adduct.

S5. The circumference of the nitrobenzene rings along the reaction path, in [Å].

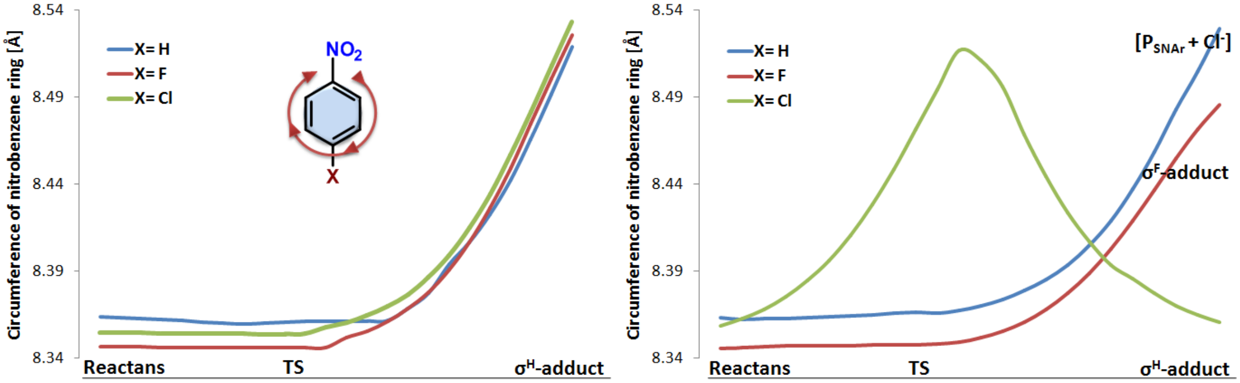

S6. The interatomic distances changes between substrates and σ^X-adducts structures in in [Å].

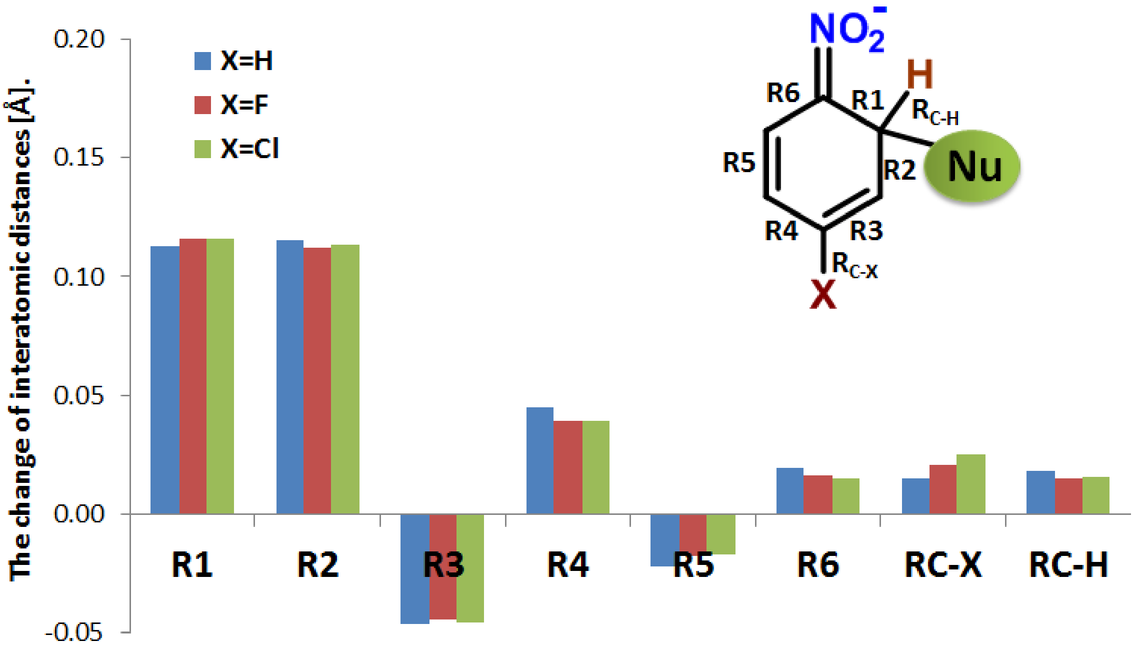

S6a. The changes of interatomic distancec between substrate and σ^H adducts.

S6b. Interatomic distances calculated at PBE1PBE/6-31+G(d) level of theory, for addition into *ortho* position in [Å].

Distance	NB	*ortho* σ^H adduct	Change	p-Fluoro NB	*ortho* σ^H adduct	Change	p-Chloro NB	*ortho* σ^H adduct	Change
R1	1.391	1.508	0.117	1.392	1.510	0.118	1.391	1.508	0.117
R2	1.391	1.506	0.115	1.389	1.504	0.116	1.389	1.507	0.118
R3	1.395	1.351	-0.044	1.389	1.343	-0.047	1.395	1.346	-0.048
R4	1.395	1.441	0.046	1.389	1.431	0.042	1.395	1.436	0.041
R5	1.391	1.368	-0.023	1.389	1.369	-0.020	1.389	1.369	-0.020
R6	1.391	1.413	0.022	1.392	1.410	0.018	1.391	1.409	0.018
RC-H	1.084	1.102	0.018	1.084	1.102	0.018	1.084	1.102	0.018
RC-X	1.087	1.102	0.016	1.340	1.365	0.025	1.731	1.762	0.031

NB- Nitrobenzene.

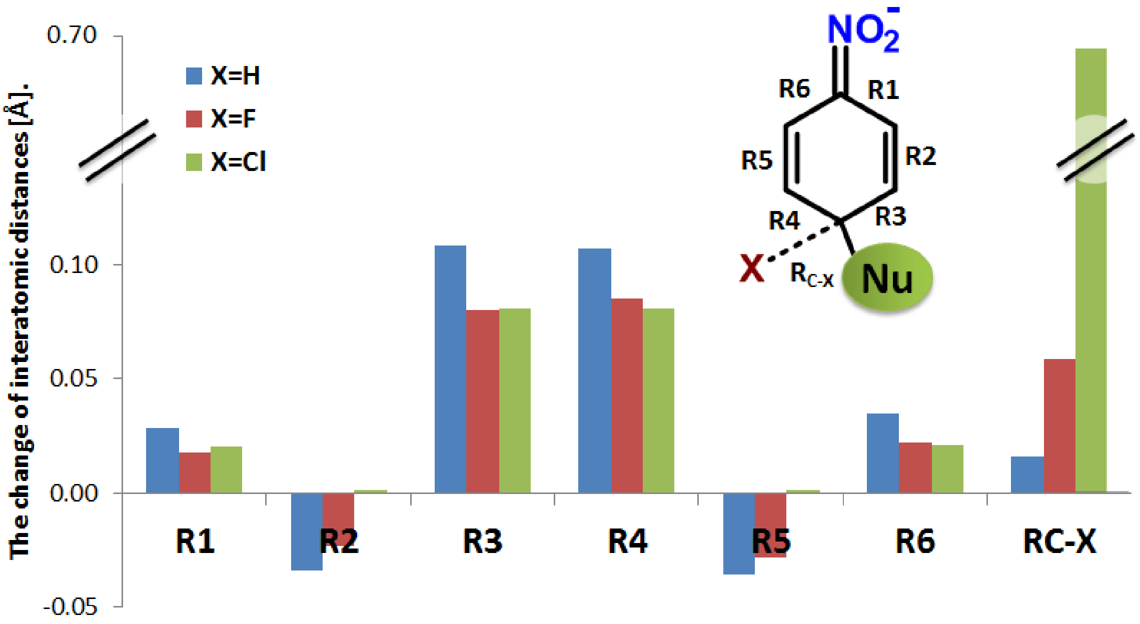

S6c. The changes of interatomic distancec between substrate and σ^X adducts.

S6d. Interatomic distances calculated at PBE1PBE/6-31+G(d) level of theory, for addition into *para* position.

Distance	NB	*para* σ^H adduct	Change	p-Fluoro-NB	*para* σ^F adduct	Change	p-Chloro NB	*para* σ^{Cl} adduct	Change
R1	1.391	1.423	0.032	1.392	1.412	0.020	1.391	1.395	0.005
R2	1.391	1.357	-0.034	1.389	1.364	-0.025	1.389	1.383	-0.006
R3	1.395	1.506	0.111	1.389	1.473	0.083	1.395	1.401	0.007
R4	1.395	1.503	0.108	1.389	1.473	0.083	1.395	1.403	0.008
R5	1.391	1.355	-0.036	1.389	1.362	-0.027	1.389	1.386	-0.003
R6	1.391	1.428	0.037	1.392	1.417	0.025	1.391	1.391	0.001
RC-X	1.087	1.105	0.018	1.340	1.454	0.114	1.731	2.376	0.645

NB- Nitrobenzene.

S7. Transition state geometries.

Geometries and energetic parameters calculated at PBE1PBE/6311+G(2d,p)|| PBE1PBE/6-31+G(d).

Transition state structures of the reaction between 4-X-nitrobenzene and carbanion of chloromethyl phenyl sulfone ($PhSO_2CHCl^-$).

Addition into *ortho* position relative to the nitro group:

X=tBu, TS1

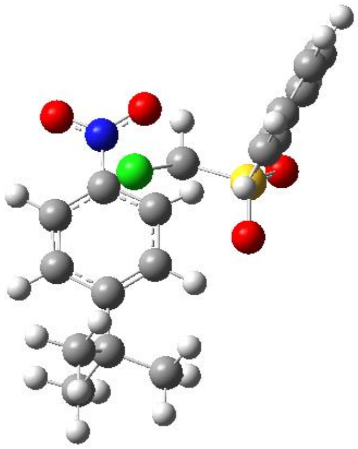

Thermochemistry:

Zero-point correction=	0.331454 (Hartree/Particle)
Thermal correction to Energy=	0.354298
Thermal correction to Enthalpy=	0.355242
Thermal correction to Gibbs Free Energy=	0.279107
Sum of electronic and zero-point Energies=	-1871.728431
Sum of electronic and thermal Energies=	-1871.705587
Sum of electronic and thermal Enthalpies=	-1871.704643
Sum of electronic and thermal Free Energies=	-1871.780778

Vibrational frequencies

**Frequency:**	**Infrared:**
-176.62	507.9739

Cartesian coordinates:

C	-1.98429100	2.00846300	-0.01053000
C	-1.32575000	1.06588600	-0.83630300
C	-1.92026800	-0.22805500	-0.91554800
C	-2.94759100	-0.62200300	-0.08870200
C	-3.50233400	0.33390500	0.81487800
C	-3.03456200	1.62448300	0.84303900
C	0.67718100	0.64482400	0.13908600
S	1.63627800	-0.36473600	-0.88682200

N	-1.55243700	3.35650000	-0.01373400
O	-2.17217100	4.19111300	0.66517600
Cl	0.43202800	0.00132600	1.76394200
O	1.73274400	0.35320900	-2.17258500
O	1.12588200	-1.74657500	-0.85617100
O	-0.56173000	3.65554200	-0.69463100
H	-0.71887500	1.42834300	-1.65639400
H	-3.46250300	2.36936800	1.50575000
H	-4.30624200	0.04927000	1.48893800
H	1.08791100	1.65324400	0.17459200
H	-1.46733400	-0.92952600	-1.60687800
C	3.32248200	-0.47294300	-0.26509100
C	3.64835600	-1.44310900	0.68335000
C	4.26771600	0.46789200	-0.67579400
C	4.93309400	-1.46864000	1.22227400
H	2.89805300	-2.17166800	0.97549300
C	5.55083000	0.43110800	-0.13681100
H	3.98815100	1.20549500	-1.42330400
C	5.88521100	-0.53433100	0.81472900
H	5.19275500	-2.22425300	1.96068500
H	6.29412400	1.15573500	-0.46251400
H	6.88793000	-0.55917200	1.23597800
C	-3.50443800	-2.04640900	-0.09992200
C	-5.00846800	-2.01817600	-0.41733200
H	-5.41848500	-3.03748100	-0.41439700
H	-5.57011800	-1.42790800	0.31523400
H	-5.18765700	-1.58006700	-1.40663200
C	-3.28805400	-2.67356400	1.28855200
H	-3.67117400	-3.70323600	1.30468000
H	-2.22121700	-2.69515900	1.53723400
H	-3.80310900	-2.10941000	2.07419300
C	-2.81119800	-2.93763300	-1.13424200
H	-2.95597000	-2.56072100	-2.15391200
H	-1.73401600	-3.01234800	-0.94898200
H	-3.23537200	-3.94926600	-1.08959000

X=tBu, TS2

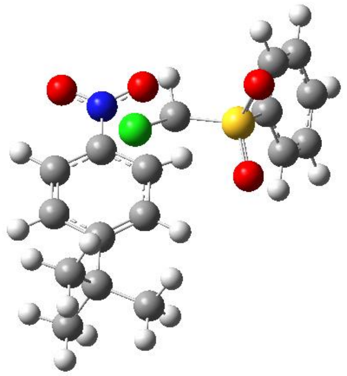

Thermochemistry:

Zero-point correction= 0.331182 (Hartree/Particle)
Thermal correction to Energy= 0.354118
Thermal correction to Enthalpy= 0.355062
Thermal correction to Gibbs Free Energy= 0.278766
Sum of electronic and zero-point Energies= -1871.719883
Sum of electronic and thermal Energies= -1871.696947
Sum of electronic and thermal Enthalpies= -1871.696003
Sum of electronic and thermal Free Energies= -1871.772299

Vibrational frequencies

**Frequency:**	**Infrared:**
**-130.53**	**221.1392**

Cartesian coordinates:

C	0.82274500	2.13096300	-0.13522600
C	0.38567400	0.91960500	0.44076400
C	1.38870800	-0.00034400	0.84161200
C	2.72097300	0.18324000	0.53513800
C	3.10689500	1.38075700	-0.13409300
C	2.17482900	2.33717700	-0.45923700
C	-0.62747400	-0.35287200	-1.29281400
S	-1.63479200	-1.68410400	-0.76762600
N	-0.12562500	3.14576700	-0.42086700
O	0.27006100	4.22522200	-0.88854400
Cl	0.71336300	-0.92921600	-2.30142300
O	-0.85076700	-2.57589200	0.11254300
O	-2.46854800	-2.33421000	-1.80511900
O	-1.32035900	2.91425000	-0.19527600
H	-0.60044800	0.88442900	0.88099900
H	2.45718400	3.26063100	-0.95377100
H	4.14788100	1.54915000	-0.39804500
H	-1.23940300	0.34911400	-1.86456700
H	1.05293300	-0.91016500	1.32650000
C	-2.78186900	-0.78290600	0.28486800
C	-3.84702200	-0.09041200	-0.28682800
C	-2.60335300	-0.80844700	1.66537100
C	-4.73126900	0.60187000	0.53607700

H	-3.98174100	-0.10647900	-1.36472200
C	-3.49939500	-0.12383100	2.48383100
H	-1.77176900	-1.37365700	2.07744100
C	-4.55876400	0.58576200	1.91988400
H	-5.55649800	1.15659400	0.09589600
H	-3.36633600	-0.13989300	3.56324700
H	-5.25059400	1.12936700	2.55940400
C	3.78906000	-0.85611500	0.87964100
C	3.21127300	-2.06740500	1.61635900
H	2.45171000	-2.58304200	1.01858300
H	4.01496000	-2.78311000	1.83265200
H	2.75611900	-1.77917200	2.57169700
C	4.43552500	-1.35618900	-0.42396800
H	4.91005500	-0.54029100	-0.98052800
H	5.20570100	-2.10775500	-0.20230100
H	3.68080000	-1.81019600	-1.07533900
C	4.86755200	-0.22065700	1.77266900
H	4.43284200	0.13281000	2.71552300
H	5.64755300	-0.95715500	2.00939600
H	5.35031500	0.63464000	1.28708600

X=tBu, TS3

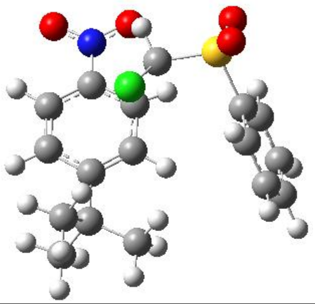

Thermochemistry:

Zero-point correction=	0.331335 (Hartree/Particle)
Thermal correction to Energy=	0.354121
Thermal correction to Enthalpy=	0.355065
Thermal correction to Gibbs Free Energy=	0.279474
Sum of electronic and zero-point Energies=	-1871.722048
Sum of electronic and thermal Energies=	-1871.699262
Sum of electronic and thermal Enthalpies=	-1871.698318
Sum of electronic and thermal Free Energies=	-1871.773909

Vibrational frequencies

**Frequency:**	**Infrared:**
-167.29	347.5079

Cartesian coordinates:

C	-1.63471200	1.94533200	-0.60595800
C	-0.62436000	0.96264000	-0.76156100
C	-1.04030100	-0.39240500	-0.60987000

C	-2.28093200	-0.73460500	-0.11467600
C	-3.20998600	0.30491800	0.17777200
C	-2.89337900	1.61861800	-0.07782500
C	0.79917400	1.23650100	1.00527000
S	2.50565700	0.92900200	0.75585100
N	-1.36181100	3.28883200	-0.96454500
O	-2.25707000	4.14026300	-0.83515100
Cl	0.12810800	0.29757600	2.34910000
O	3.27965200	0.58372700	1.96472700
O	2.99220300	2.00409500	-0.12780200
O	-0.23695600	3.56371800	-1.39780600
H	0.22222500	1.20177700	-1.39161100
H	-3.60577000	2.41809500	0.09704700
H	-4.18874200	0.06814700	0.58677000
H	0.68207500	2.30664300	1.18271000
H	-0.31105600	-1.16062600	-0.84440300
C	2.51972700	-0.57288200	-0.23378300
C	2.56631800	-0.47154500	-1.62264900
C	2.48247700	-1.81773800	0.39233400
C	2.56269000	-1.63195900	-2.39399100
H	2.61847800	0.51393200	-2.07763400
C	2.47547800	-2.97351800	-0.38609700
H	2.47242200	-1.86710500	1.47719600
C	2.51230500	-2.88266900	-1.77785600
H	2.60006100	-1.55912100	-3.47863600
H	2.44446000	-3.94850300	0.09541600
H	2.50680200	-3.78721800	-2.38225500
C	-2.70119700	-2.18705700	0.12101400
C	-3.02364100	-2.38026000	1.61279600
H	-3.84507400	-1.73267700	1.93833100
H	-3.31667800	-3.42120800	1.80761800
H	-2.14917800	-2.14285400	2.22858300
C	-1.60620700	-3.18685100	-0.25904800
H	-1.95868600	-4.20856900	-0.06650400
H	-1.34347400	-3.11891200	-1.32137100
H	-0.69354300	-3.02896700	0.32585400
C	-3.95136200	-2.50801000	-0.71515700
H	-3.74494800	-2.38390800	-1.78496600
H	-4.26864100	-3.54590100	-0.54432100
H	-4.79158500	-1.85316000	-0.46035000

X=OCH_3, TS1

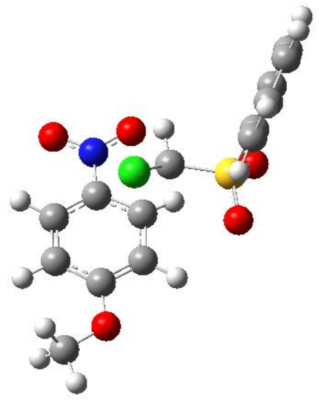

Thermochemistry:

Zero-point correction= 0.251887 (Hartree/Particle)
Thermal correction to Energy= 0.271933
Thermal correction to Enthalpy= 0.272877
Thermal correction to Gibbs Free Energy= 0.201998
Sum of electronic and zero-point Energies= -1829.136490
Sum of electronic and thermal Energies= -1829.116444
Sum of electronic and thermal Enthalpies= -1829.115500
Sum of electronic and thermal Free Energies= -1829.186379

Vibrational frequencies

Frequency: Infrared:
-177.56 541.8808

Cartesian coordinates:

C	2.46638700	1.41866800	-0.04418400
C	1.70619800	0.62370400	0.86134900
C	2.17596800	-0.69571800	1.07964600
C	3.17384800	-1.23503300	0.29638600
C	3.83551800	-0.45996200	-0.69118500
C	3.47555600	0.86223000	-0.84014200
C	-0.30743500	0.36464400	-0.14600200
S	-1.46327600	-0.24503100	0.98782800
N	2.17212000	2.79729800	-0.17833300
O	2.86260200	3.49249900	-0.94193100
Cl	-0.10020900	-0.64350300	-1.58113400
O	-1.51618700	0.75079100	2.07543900
O	-1.17169300	-1.65429200	1.29863200
O	1.22795700	3.26156400	0.47240700
H	1.13323000	1.12644200	1.62983900
H	3.97449500	1.49961500	-1.56232700
H	4.61267000	-0.88101400	-1.31904100
H	-0.55123200	1.39071500	-0.41925900
H	1.68387100	-1.32744000	1.81237100
C	-3.11178700	-0.25629200	0.26159800
C	-3.53370800	-1.36246000	-0.47665300
C	-3.92210400	0.87386000	0.37715000

C	-4.77842500	-1.33324300	-1.10241500
H	-2.88959800	-2.23455700	-0.53865100
C	-5.16712900	0.89214200	-0.24577900
H	-3.57100300	1.71621000	0.96733900
C	-5.59646800	-0.20909900	-0.98898600
H	-5.11278300	-2.19415500	-1.67759500
H	-5.80679000	1.76683800	-0.14812000
H	-6.56880900	-0.19129400	-1.47670300
O	3.50476800	-2.53573900	0.55574500
C	4.49654200	-3.14089800	-0.23124700
H	4.58188700	-4.16857000	0.13079700
H	4.21862300	-3.15755700	-1.29433100
H	5.46923400	-2.64011600	-0.12151400

X=OCH3, TS2

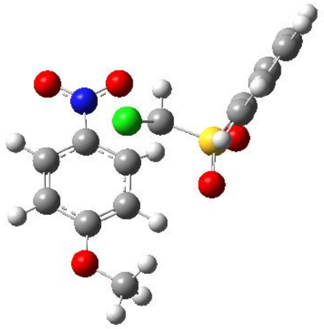

Thermochemistry:

Zero-point correction= 0.252240 (Hartree/Particle)
Thermal correction to Energy= 0.272177
Thermal correction to Enthalpy= 0.273121
Thermal correction to Gibbs Free Energy= 0.202653
Sum of electronic and zero-point Energies= -1829.141437
Sum of electronic and thermal Energies= -1829.121499
Sum of electronic and thermal Enthalpies= -1829.120555
Sum of electronic and thermal Free Energies= -1829.191024

Vibrational frequencies

**Frequency:**	**Infrared:**
**-185.09**	**539.4741**

Cartesian coordinates:

C	2.67211600	-1.27752100	-0.03556200
C	1.84370100	-0.43950100	-0.82739000
C	2.15937600	0.94814400	-0.83309100
C	3.07567500	1.46086900	0.05685100
C	3.82475100	0.61234900	0.91636700
C	3.62081100	-0.74252300	0.85698000
C	-0.20896100	-0.52428200	0.12214700
S	-1.32397400	0.37202000	-0.84789000
N	2.51668800	-2.67923400	-0.11622000

O	3.27659500	-3.41213500	0.53859600
Cl	-0.09806500	0.02702100	1.79469700
O	-1.29145200	-0.26188900	-2.18055900
O	-1.05603200	1.81660200	-0.72168500
O	1.61794000	-3.13197600	-0.83958700
H	1.33957700	-0.87269700	-1.68171400
H	4.18288200	-1.42633900	1.48401500
H	4.53679600	1.05675600	1.60498200
H	-0.42933300	-1.58995400	0.07237100
H	1.57958800	1.59382400	-1.48159800
C	-3.00816100	0.15564500	-0.24845300
C	-3.49732000	0.99507200	0.75291600
C	-3.77899100	-0.90315100	-0.73008100
C	-4.77062000	0.77071600	1.27250900
H	-2.88131800	1.81856300	1.10159700
C	-5.05239300	-1.11661700	-0.20936700
H	-3.37530200	-1.53460300	-1.51706900
C	-5.54957300	-0.28271000	0.79411700
H	-5.15722400	1.42360700	2.05219200
H	-5.66114300	-1.93397900	-0.59019000
H	-6.54404100	-0.45348500	1.20078300
O	3.36105700	2.78749900	0.17729800
C	2.55455700	3.68807300	-0.54978000
H	2.86045600	4.68817400	-0.23277700
H	2.71797800	3.58616000	-1.63163700
H	1.49027500	3.53475000	-0.33249800

X=OCH_3, TS3

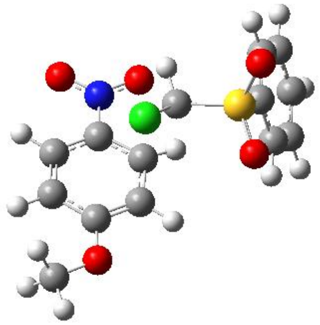

Thermochemistry:

Zero-point correction= 0.251607 (Hartree/Particle)
Thermal correction to Energy= 0.271759
Thermal correction to Enthalpy= 0.272703
Thermal correction to Gibbs Free Energy= 0.201553
Sum of electronic and zero-point Energies= -1829.128474
Sum of electronic and thermal Energies= -1829.108322
Sum of electronic and thermal Enthalpies= -1829.107378
Sum of electronic and thermal Free Energies= -1829.178527

**Vibrational frequencies**			
**Frequency:**	**Infrared:**		
**-117.11**	**208.7721**		
**Cartesian coordinates:**			
C	1.40056800	1.73000700	-0.23119800
C	0.81989100	0.69529800	0.54616400
C	1.70485900	-0.19059100	1.19090000
C	3.06075900	-0.15936400	0.92288400
C	3.61335500	0.81842300	0.06088500
C	2.77225400	1.75621500	-0.50354100
C	-0.23220200	-0.73033500	-1.06777900
S	-1.54974800	-1.74702700	-0.52583000
N	0.56815700	2.73904000	-0.78116100
O	1.08402000	3.65019400	-1.44783600
Cl	1.10410200	-1.70355500	-1.71417700
O	-1.06817100	-2.65906200	0.53082400
O	-2.40788400	-2.33258900	-1.58315900
O	-0.64872100	2.66872400	-0.57630600
H	-0.19022200	0.82379400	0.90568400
H	3.15863000	2.53442600	-1.15305700
H	4.67387000	0.84407600	-0.16305000
H	-0.60822900	-0.06525700	-1.84948100
H	1.31728500	-0.96931100	1.84056700
C	-2.56963900	-0.50474200	0.28354900
C	-3.43314800	0.28618500	-0.47077700
C	-2.49736200	-0.36525600	1.66733900
C	-4.21913100	1.24091200	0.16883100
H	-3.49027100	0.14267100	-1.54599700
C	-3.29514600	0.58421500	2.30230400
H	-1.82648000	-1.01242100	2.22597700
C	-4.15150200	1.39086100	1.55362000
H	-4.88423900	1.87191300	-0.41606400
H	-3.24446300	0.69743000	3.38305400
H	-4.76564500	2.13947300	2.04933700
O	3.81979200	-1.09467900	1.56394900
C	5.19321000	-1.14884300	1.27680100
H	5.59007900	-1.98035300	1.86427300
H	5.37675600	-1.34195300	0.21101800
H	5.70918800	-0.22350100	1.57048300

**X=OCH_3, TS4**

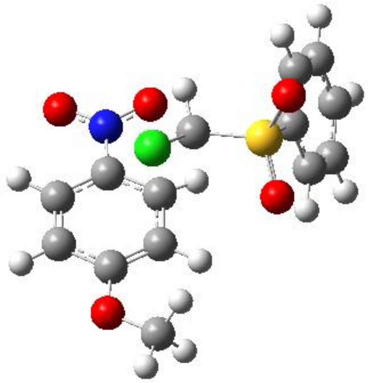

Thermochemistry:

Zero-point correction= 0.251968 (Hartree/Particle)
Thermal correction to Energy= 0.271995
Thermal correction to Enthalpy= 0.272939
Thermal correction to Gibbs Free Energy= 0.202290
Sum of electronic and zero-point Energies= -1829.132994
Sum of electronic and thermal Energies= -1829.112967
Sum of electronic and thermal Enthalpies= -1829.112023
Sum of electronic and thermal Free Energies= -1829.182672

Vibrational frequencies

Frequency: Infrared:
-135.57 225.6838

Cartesian coordinates:

C	1.50100800	1.86575800	-0.01602500
C	0.87892900	0.73738500	0.56757300
C	1.72074900	-0.27426000	1.08840300
C	3.08082200	-0.23314200	0.85913900
C	3.68294600	0.87289400	0.20571000
C	2.89134800	1.90808700	-0.22597000
C	-0.14375400	-0.47505000	-1.20230900
S	-1.33706100	-1.64462000	-0.68777700
N	0.70307300	2.95956400	-0.42705700
O	1.25491700	3.96200700	-0.90994800
Cl	1.21051500	-1.27168200	-2.02683400
O	-0.74137400	-2.53403800	0.33355200
O	-2.13836300	-2.29688800	-1.74935000
O	-0.52495500	2.87507500	-0.29375100
H	-0.13532700	0.84056300	0.92459800
H	3.31510600	2.77282800	-0.72530700
H	4.75670400	0.86732900	0.04522200
H	-0.61601600	0.24057400	-1.87962600
H	1.25048000	-1.12939100	1.55993800
C	-2.47012600	-0.54398300	0.17278600
C	-3.37706900	0.22299800	-0.55544000
C	-2.44240400	-0.49013000	1.56358300

C	-4.25044800	1.07096900	0.11982000
H	-3.39954700	0.14433300	-1.63876400
C	-3.32816900	0.35124700	2.23374300
H	-1.73514700	-1.11734800	2.09941500
C	-4.22677500	1.13620800	1.51280800
H	-4.95066900	1.68388500	-0.44276400
H	-3.31169800	0.39856100	3.32031200
H	-4.90902600	1.80204300	2.03655800
O	3.94735000	-1.20969800	1.24365500
C	3.39149300	-2.40063000	1.75625800
H	4.22989800	-3.08728500	1.89583900
H	2.90024000	-2.22857400	2.72392700
H	2.66581700	-2.83372800	1.05692300

X=OCH$_3$, TS5

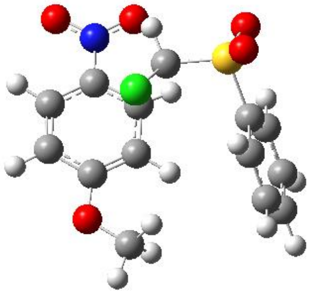

Thermochemistry:

Zero-point correction= 0.252069 (Hartree/Particle)
Thermal correction to Energy= 0.271957
Thermal correction to Enthalpy= 0.272901
Thermal correction to Gibbs Free Energy= 0.202906
Sum of electronic and zero-point Energies= -1829.135258
Sum of electronic and thermal Energies= -1829.115370
Sum of electronic and thermal Enthalpies= -1829.114426
Sum of electronic and thermal Free Energies= -1829.184421

Vibrational frequencies

**Frequency:**	**Infrared:**
**-166.47**	**342.2858**

Cartesian coordinates:

C	2.59655700	-0.13770100	-0.54898800
C	1.19918100	0.03589200	-0.74017100
C	0.68242200	1.33839300	-0.49902500
C	1.46169700	2.29784500	0.11317500
C	2.82411600	2.05493800	0.42213900
C	3.37836400	0.84605900	0.07976900
C	0.21155700	-1.22872600	0.89716800
S	-1.35087900	-1.94020500	0.56930300

N	3.21223500	-1.33768500	-0.97514400
O	4.43644800	-1.47832900	-0.81359500
Cl	0.20741100	-0.21408600	2.34875400
O	-2.17928000	-2.26229800	1.74854300
O	-1.12606700	-2.97705600	-0.45489500
O	2.50512000	-2.20756100	-1.49799400
H	0.69961700	-0.62061100	-1.44028300
H	4.42506900	0.63204700	0.26780400
H	3.39969400	2.83299500	0.91381500
H	0.93391900	-2.04122400	0.98710900
H	-0.35662200	1.52944000	-0.74261000
C	-2.24280200	-0.61289700	-0.25524800
C	-2.24842700	-0.55947500	-1.64761300
C	-2.91303700	0.34821000	0.50008500
C	-2.92209700	0.47700200	-2.29078900
H	-1.73480100	-1.33769100	-2.20568100
C	-3.58118400	1.38427400	-0.15006600
H	-2.91418800	0.26720100	1.58306500
C	-3.58381500	1.45244100	-1.54391800
H	-2.93116300	0.52271200	-3.37752800
H	-4.10761600	2.13699200	0.43314600
H	-4.10875200	2.26090100	-2.04846900
O	1.02207900	3.54595600	0.43992000
C	-0.34261900	3.82749700	0.23998500
H	-0.50142300	4.83801300	0.62471000
H	-0.60794100	3.79898500	-0.82587000
H	-0.97923800	3.11856000	0.78429300

X=NO_2, TS1

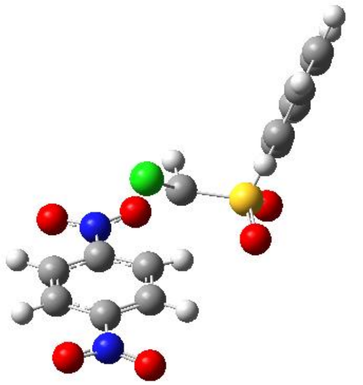

Thermochemistry:

Zero-point correction= 0.222146 (Hartree/Particle)
Thermal correction to Energy= 0.242099
Thermal correction to Enthalpy= 0.243043
Thermal correction to Gibbs Free Energy= 0.171886
Sum of electronic and zero-point Energies= -1919.109256

Sum of electronic and thermal Energies= -1919.089303
Sum of electronic and thermal Enthalpies= -1919.088359
Sum of electronic and thermal Free Energies= -1919.159516

Vibrational frequencies

Frequency: Infrared:
-46.40 75.3659

Cartesian coordinates:

C	-2.54200500	1.55769300	0.03525500
C	-1.71580000	0.77371500	-0.79092100
C	-2.03539800	-0.59768700	-0.90538700
C	-3.02949300	-1.12740400	-0.11514200
C	-3.78927200	-0.35527800	0.79053100
C	-3.53789700	0.99422100	0.85272300
C	0.45805900	0.47738300	0.31245900
S	1.55441100	-0.06027700	-0.90309200
N	-2.33519000	2.97176600	0.08163200
O	-3.09001900	3.65682900	0.78264100
Cl	0.27942600	-0.59387200	1.69723400
O	1.57366900	1.01188000	-1.91697300
O	1.22131500	-1.43890900	-1.29912600
O	-1.40893500	3.44601300	-0.57739200
H	-1.06370200	1.25157500	-1.50958100
H	-4.10309700	1.64451500	1.51073500
H	-4.54611300	-0.82846800	1.40342700
H	0.67194000	1.50037700	0.61767200
H	-1.45681800	-1.23738200	-1.56250700
C	3.23163300	-0.15193600	-0.25661000
C	3.66314800	-1.31792700	0.37600500
C	4.05785600	0.97010600	-0.32646500
C	4.93570300	-1.35709300	0.94239800
H	3.00571300	-2.18191000	0.40210000
C	5.33014800	0.91997300	0.23676300
H	3.69838100	1.85979300	-0.83660400
C	5.76998200	-0.24145900	0.87441600
H	5.27862400	-2.26475300	1.43424400
H	5.98290700	1.78803100	0.17396500
H	6.76411000	-0.27731200	1.31468000
N	-3.33093500	-2.55247800	-0.23064300
O	-2.78033700	-3.19756800	-1.11118600
O	-4.13692700	-3.02762800	0.56394700

X=NO_2, TS2

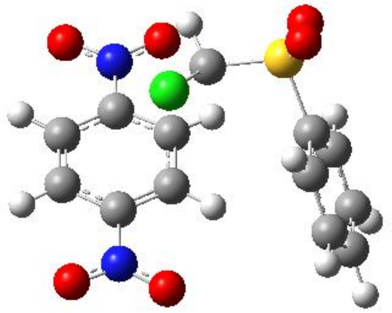

Thermochemistry:

Zero-point correction= 0.221965 (Hartree/Particle)
Thermal correction to Energy= 0.241920
Thermal correction to Enthalpy= 0.242865
Thermal correction to Gibbs Free Energy= 0.172013
Sum of electronic and zero-point Energies= -1919.104243
Sum of electronic and thermal Energies= -1919.084288
Sum of electronic and thermal Enthalpies= -1919.083344
Sum of electronic and thermal Free Energies= -1919.154195

Vibrational frequencies

**Frequency:**	**Infrared:**
**-30.71**	**43.0824**

Cartesian coordinates:

C	2.46823700	-0.85622000	-0.61100200
C	1.14524200	-0.43166200	-0.83092300
C	0.85952400	0.92835700	-0.60477800
C	1.81861800	1.73597800	-0.03413100
C	3.11172100	1.27836300	0.29272000
C	3.42855900	-0.02576200	-0.00958900
C	-0.25763100	-1.30149700	1.07005800
S	-1.93670100	-1.64076200	0.76295200
N	2.84278800	-2.18598700	-0.98437800
O	4.00784900	-2.54840700	-0.78264600
Cl	0.02192900	-0.18745900	2.41034500
O	-2.82639800	-1.59156800	1.93901500
O	-1.95673600	-2.83218700	-0.10769500
O	1.98678900	-2.90912200	-1.49456800
H	0.46861400	-1.05625700	-1.39620000
H	4.41535800	-0.42764800	0.19046400
H	3.82297400	1.94985500	0.75695100
H	0.27247600	-2.24344900	1.21152500
H	-0.12146100	1.32899100	-0.83253200
C	-2.45290300	-0.26055200	-0.26451100
C	-2.45719600	-0.40780900	-1.65040200
C	-2.81049800	0.94979700	0.32847700
C	-2.81056200	0.67617500	-2.45177300
H	-2.19254200	-1.37098700	-2.07857700

C	-3.15054400	2.03167600	-0.48048300
H	-2.81629200	1.03380200	1.41111600
C	-3.14932700	1.89769200	-1.86897900
H	-2.81717600	0.56780000	-3.53413300
H	-3.40939600	2.98444800	-0.02525700
H	-3.41011500	2.74672200	-2.49664400
N	1.49120000	3.13534700	0.22389700
O	0.41185900	3.56527800	-0.16129200
O	2.32721600	3.81513100	0.81036700

X=NO_2, TS3

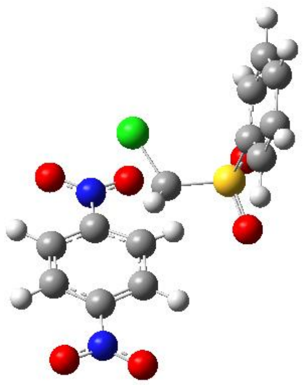

Thermochemistry:

Zero-point correction= 0.222122 (Hartree/Particle)
Thermal correction to Energy= 0.242093
Thermal correction to Enthalpy= 0.243038
Thermal correction to Gibbs Free Energy= 0.171878
Sum of electronic and zero-point Energies= -1919.108753
Sum of electronic and thermal Energies= -1919.088781
Sum of electronic and thermal Enthalpies= -1919.087837
Sum of electronic and thermal Free Energies= -1919.158996

Vibrational frequencies

**Frequency:**	**Infrared:**
**-47.73**	**90.0421**

Cartesian coordinates:

C	-2.52127200	-1.32028900	0.02538500
C	-1.79436300	-0.46009500	0.86488400
C	-2.12788900	0.91174900	0.84069300
C	-3.00065200	1.37518200	-0.11836600
C	-3.60326900	0.52616300	-1.07469100
C	-3.36510600	-0.82554400	-0.98409900
C	0.37280600	-0.16937200	-0.23372200
S	1.57489500	0.07671400	0.97645900
N	-2.40780700	-2.74042500	0.20228700
O	-3.00133600	-3.47581500	-0.59349300
O	1.72544200	-1.13251700	1.79803800

O	1.22337900	1.36416300	1.60923200
O	-1.75962700	-3.15912400	1.15843600
H	-1.21622400	-0.87383800	1.68041100
H	-3.83890500	-1.52743200	-1.66101600
H	-4.25943500	0.94282700	-1.82872300
H	-1.64933500	1.59811700	1.53015100
C	3.18610800	0.33594600	0.21644200
C	3.59545600	1.62923300	-0.10902100
C	3.97874400	-0.76570900	-0.10694700
C	4.81165700	1.81984500	-0.75976800
H	2.96394800	2.46873900	0.16915600
C	5.19098800	-0.56621200	-0.76414500
H	3.64463500	-1.76054300	0.17260100
C	5.60924200	0.72351700	-1.09189600
H	5.14079500	2.82751200	-1.00440500
H	5.81365200	-1.42171900	-1.01659300
H	6.55798100	0.87519400	-1.60203800
Cl	0.53374100	-1.60550300	-1.22493500
H	0.24524400	0.72427000	-0.84282700
N	-3.31452900	2.79626900	-0.15318300
O	-4.03456400	3.19853900	-1.06359500
O	-2.85486900	3.52170600	0.72033400

X=Br, TS1

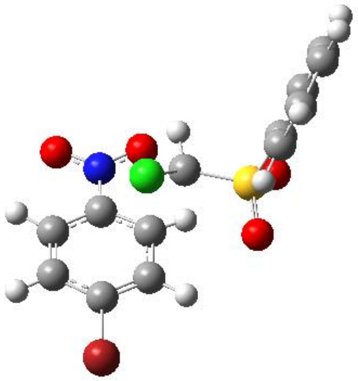

Thermochemistry:

Zero-point correction= 0.209343 (Hartree/Particle)
Thermal correction to Energy= 0.228338
Thermal correction to Enthalpy= 0.229282
Thermal correction to Gibbs Free Energy= 0.159562
Sum of electronic and zero-point Energies= -4287.970103
Sum of electronic and thermal Energies= -4287.951108
Sum of electronic and thermal Enthalpies= -4287.950164
Sum of electronic and thermal Free Energies= -4288.019884

Vibrational frequencies

Frequency: Infrared:

**-96.92**	**249.0732**		
**Cartesian coordinates:**			
C	-2.06211000	1.97726200	0.07193100
C	-1.37044800	1.08911600	-0.78033800
C	-1.88471600	-0.22795800	-0.88345300
C	-2.90857200	-0.62409100	-0.06025500
C	-3.52831400	0.24361700	0.86326900
C	-3.09569000	1.54945700	0.91857500
C	0.72784200	0.62887600	0.25506800
S	1.76020800	-0.06634500	-0.93728900
N	-1.68247900	3.35055000	0.09694500
O	-2.31159300	4.13112100	0.82530100
Cl	0.41809800	-0.39789200	1.65561700
O	1.92835000	0.97103700	-1.97324000
O	1.26092300	-1.40087800	-1.31322900
O	-0.73596600	3.70877800	-0.60785500
H	-0.72488800	1.48917900	-1.55055300
H	-3.54968100	2.26659700	1.59371300
H	-4.32445200	-0.11485100	1.50666000
H	1.08176200	1.61511600	0.55198900
H	-1.41056900	-0.92881000	-1.56201000
C	3.40759500	-0.35863300	-0.27142200
C	3.67672900	-1.54708700	0.40807600
C	4.37711000	0.63976600	-0.37180300
C	4.92985400	-1.73472600	0.98793900
H	2.90968500	-2.31402700	0.45848900
C	5.62820600	0.44197800	0.20635000
H	4.14227300	1.55003600	-0.91711300
C	5.90615700	-0.74338800	0.88947700
H	5.14587100	-2.66118300	1.51581500
H	6.39106900	1.21289000	0.11967200
H	6.88415300	-0.89516600	1.34122600
Br	-3.54269800	-2.40381500	-0.16381900

X=Br, TS2

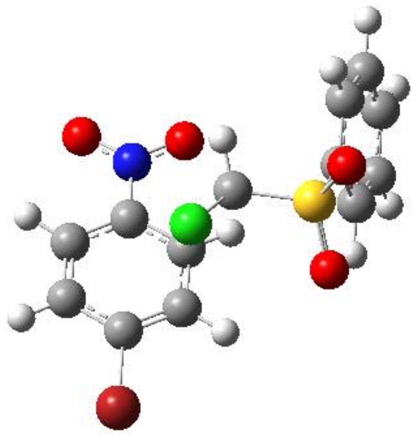

Thermochemistry:

Zero-point correction= 0.209079 (Hartree/Particle)
Thermal correction to Energy= 0.228153
Thermal correction to Enthalpy= 0.229097
Thermal correction to Gibbs Free Energy= 0.159246
Sum of electronic and zero-point Energies= -4287.960987
Sum of electronic and thermal Energies= -4287.941913
Sum of electronic and thermal Enthalpies= -4287.940969
Sum of electronic and thermal Free Energies= -4288.010820

Vibrational frequencies

**Frequency:**	**Infrared:**
**-41.64**	**55.4197**

Cartesian coordinates:

C	0.76002600	2.13461800	-0.21652900
C	0.42989800	0.95189300	0.46787800
C	1.48193600	0.08917300	0.83161300
C	2.75756700	0.36169500	0.39410200
C	3.07422500	1.50746200	-0.35959700
C	2.06260600	2.39363400	-0.66342300
C	-0.61862600	-0.68244600	-1.18152100
S	-1.63023900	-1.87782300	-0.41129700
N	-0.26616400	3.09083700	-0.48826100
O	0.03668000	4.13346500	-1.08303100
Cl	0.80290000	-1.43141500	-1.93875300
O	-0.90267500	-2.47485400	0.72940200
O	-2.35904400	-2.81721500	-1.29596900
O	-1.41421500	2.83625700	-0.12048700
H	-0.55148000	0.84891800	0.90512300
H	2.25507500	3.29795800	-1.23019700
H	4.09131100	1.68071600	-0.69448000
H	-1.20384600	-0.15877500	-1.94215900
H	1.25794200	-0.82134500	1.37721500
C	-2.88215500	-0.78518400	0.27930600
C	-3.90634500	-0.30564200	-0.53436200

C	-2.82732900	-0.45321100	1.63018000
C	-4.86978100	0.54063500	0.00802500
H	-3.94882700	-0.60745500	-1.57734500
C	-3.80220800	0.38339100	2.16949200
H	-2.02947200	-0.86732900	2.24102100
C	-4.81797800	0.88646300	1.35812400
H	-5.66473400	0.92990200	-0.62390100
H	-3.76391600	0.64880600	3.22361600
H	-5.57064500	1.55014900	1.77778700
Br	4.15683700	-0.83755100	0.81604800

X=Br, TS3

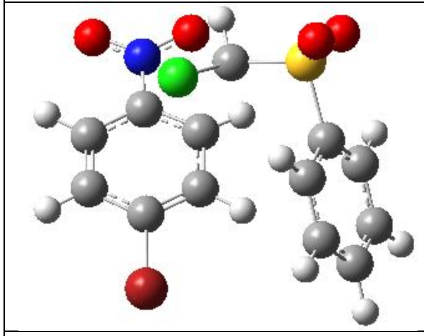

Thermochemistry:

Zero-point correction= 0.209294 (Hartree/Particle)
Thermal correction to Energy= 0.228230
Thermal correction to Enthalpy= 0.229174
Thermal correction to Gibbs Free Energy= 0.160042
Sum of electronic and zero-point Energies= -4287.964409
Sum of electronic and thermal Energies= -4287.945473
Sum of electronic and thermal Enthalpies= -4287.944529
Sum of electronic and thermal Free Energies= -4288.013662

Vibrational frequencies

**Frequency:**	**Infrared:**
**-90.37**	**176.2085**

Cartesian coordinates:

C	2.24802700	-1.32158500	-0.56697800
C	0.98575900	-0.79195000	-0.91265500
C	0.80871200	0.59857500	-0.71787200
C	1.76383800	1.32316800	-0.04705200
C	2.97623800	0.75906600	0.39412800
C	3.21153900	-0.57086000	0.11982900
C	-0.51128500	-1.73964200	0.74727100
S	-2.23011400	-1.69370100	0.48739600
N	2.55350400	-2.67094600	-0.91676100
O	3.66817900	-3.12444700	-0.62569700
Cl	0.00063900	-0.95920800	2.25273700
O	-3.07590800	-1.71074100	1.69751900

O	-2.50656100	-2.66332600	-0.59239900
O	1.69047800	-3.33195200	-1.49849500
H	0.35022800	-1.33827300	-1.59534200
H	4.14004500	-1.05079200	0.40875700
H	3.70394700	1.36097900	0.92751900
H	-0.18745200	-2.78041700	0.71108000
H	-0.09961100	1.07803800	-1.06411500
C	-2.49323800	-0.06620700	-0.22937300
C	-2.65189000	0.04896800	-1.60890500
C	-2.50344800	1.06350600	0.58875600
C	-2.81168500	1.31126600	-2.17773500
H	-2.64907700	-0.85423700	-2.21301700
C	-2.64868900	2.32250800	0.01046700
H	-2.39235600	0.95091200	1.66296800
C	-2.80196100	2.44931500	-1.37045800
H	-2.93691400	1.40734600	-3.25414400
H	-2.63556400	3.20897900	0.64014700
H	-2.91198300	3.43497800	-1.81713800
Br	1.47926200	3.17176600	0.24833600

X=Br, TS4

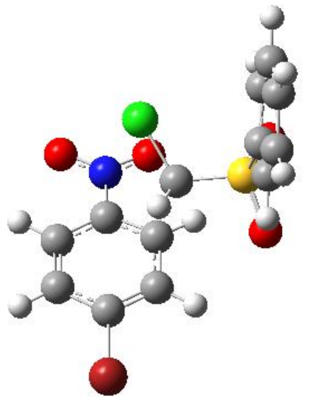

Thermochemistry:

Zero-point correction= 0.209360 (Hartree/Particle)
Thermal correction to Energy= 0.228367
Thermal correction to Enthalpy= 0.229312
Thermal correction to Gibbs Free Energy= 0.159484
Sum of electronic and zero-point Energies= -4287.968580
Sum of electronic and thermal Energies= -4287.949573
Sum of electronic and thermal Enthalpies= -4287.948628
Sum of electronic and thermal Free Energies= -4288.018456

Vibrational frequencies

Frequency:	Infrared:
**-68.88**	**185.5467**

Cartesian coordinates:

C	2.16884300	1.75762900	-0.00898800

C	1.52317400	0.86905700	0.87163200
C	1.97696600	-0.47288400	0.88911200
C	2.84932200	-0.89850800	-0.08124500
C	3.36595900	-0.03680100	-1.07044600
C	3.02777500	1.29987100	-1.01602500
C	-0.60540100	0.44718700	-0.17250100
S	-1.79515500	0.08271700	1.01865600
N	1.94415800	3.16651300	0.12100200
O	2.46450600	3.92377100	-0.70682900
O	-2.18127400	1.29687800	1.75188300
O	-1.26046000	-1.08414300	1.75314500
O	1.27188800	3.56613300	1.06954300
H	0.95311100	1.26592800	1.70035400
H	3.43668400	2.01372900	-1.72264900
H	4.03758400	-0.41540900	-1.83357100
H	1.55994000	-1.16181100	1.61582500
C	-3.31564500	-0.49951800	0.24593400
C	-3.49729600	-1.86369600	0.01660300
C	-4.26583300	0.42655000	-0.18638500
C	-4.64180500	-2.30306500	-0.64389800
H	-2.74759600	-2.56271000	0.37766000
C	-5.40434600	-0.02062200	-0.85364200
H	-4.10913100	1.48158300	0.01791800
C	-5.59557300	-1.38325800	-1.08321500
H	-4.79310600	-3.36737200	-0.81223600
H	-6.14824200	0.69820500	-1.19056300
H	-6.48796500	-1.72888800	-1.60065200
Cl	-0.98966000	1.77387500	-1.26561200
H	-0.34450900	-0.45089300	-0.73188900
Br	3.39162000	-2.71343100	-0.10953900

X=CF_3, TS1

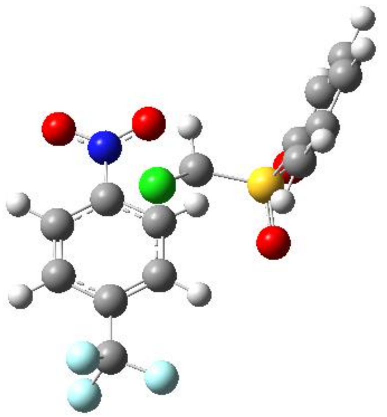

Thermochemistry:

Zero-point correction=	0.224024 (Hartree/Particle)

Thermal correction to Energy= 0.245085<br>Thermal correction to Enthalpy= 0.246030<br>Thermal correction to Gibbs Free Energy= 0.172341<br>Sum of electronic and zero-point Energies= -2051.581394<br>Sum of electronic and thermal Energies= -2051.560332<br>Sum of electronic and thermal Enthalpies= -2051.559388<br>Sum of electronic and thermal Free Energies= -2051.633077
**Vibrational frequencies**
**Frequency: Infrared:**<br>**-67.07 142.6153**
**Cartesian coordinates:**

C	-2.13900900	1.93380600	0.05312100
C	-1.41891600	1.05125200	-0.77422600
C	-1.90611100	-0.27638300	-0.87018200
C	-2.94998600	-0.69529300	-0.07792200
C	-3.59881000	0.18736900	0.82033400
C	-3.18889700	1.49790200	0.87912700
C	0.71017700	0.59126600	0.28690900
S	1.73217300	-0.10709200	-0.91087500
N	-1.76872900	3.31336100	0.08631500
O	-2.43246000	4.08917400	0.78637800
Cl	0.44455800	-0.40463400	1.71678500
O	1.84630400	0.90901700	-1.97545500
O	1.26092600	-1.46334100	-1.24170100
O	-0.79765300	3.67209400	-0.58209200
H	-0.74511200	1.44881400	-1.52086900
H	-3.66719400	2.21415500	1.53783900
H	-4.40980800	-0.16916800	1.44811600
H	1.04324900	1.59350800	0.55222300
H	-1.39816400	-0.97103400	-1.53080600
C	3.40284300	-0.33679900	-0.27925800
C	3.72218800	-1.49995000	0.42186400
C	4.33987100	0.68648100	-0.42615500
C	4.99193700	-1.63560100	0.97970600
H	2.97970400	-2.28768100	0.50813100
C	5.60851600	0.53974300	0.12849600
H	4.06601300	1.57548500	-0.98804900
C	5.93587200	-0.61896000	0.83506400
H	5.24648500	-2.54166100	1.52543600
H	6.34619900	1.32996200	0.00558500
H	6.92720600	-0.73031000	1.26910300
C	-3.45712000	-2.10116200	-0.16041200
F	-4.75442500	-2.14358800	-0.56801100
F	-3.43674700	-2.72085200	1.04327300
F	-2.76571500	-2.87566500	-1.01060600

X=CF_3, TS2

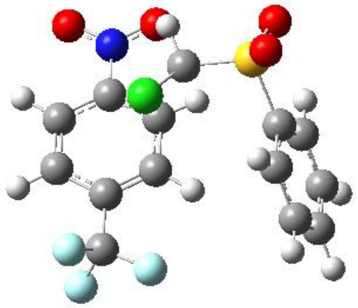

Thermochemistry:

Zero-point correction= 0.223878 (Hartree/Particle)
Thermal correction to Energy= 0.244917
Thermal correction to Enthalpy= 0.245862
Thermal correction to Gibbs Free Energy= 0.172523
Sum of electronic and zero-point Energies= -2051.576167
Sum of electronic and thermal Energies= -2051.555127
Sum of electronic and thermal Enthalpies= -2051.554183
Sum of electronic and thermal Free Energies= -2051.627521

Vibrational frequencies

Frequency: Infrared:
-50.19 92.8360

Cartesian coordinates:

C	-1.83110200	1.81428100	-0.60156600
C	-0.75265000	0.93381600	-0.80895100
C	-1.00133900	-0.44163900	-0.60010200
C	-2.19857300	-0.86156600	-0.06328900
C	-3.23017900	0.05509100	0.24522400
C	-3.04071300	1.39015100	-0.03133900
C	0.83526100	1.29237500	1.05914700
S	2.52695700	1.03332500	0.75005200
N	-1.68506600	3.19117500	-0.96157300
O	-2.63877300	3.95647400	-0.77158500
Cl	0.19877800	0.32306700	2.39425800
O	3.35540400	0.67476000	1.91826200
O	2.95862700	2.14229400	-0.12327400
O	-0.61440700	3.55398800	-1.44927500
H	0.10026800	1.26527300	-1.38308300
H	-3.81396500	2.12531100	0.16183500
H	-4.16017000	-0.29353600	0.68387900
H	0.67008600	2.35741000	1.22407900
H	-0.21883500	-1.15859200	-0.82441700
C	2.53161800	-0.44130600	-0.27905000
C	2.56625100	-0.30446900	-1.66561700
C	2.47554200	-1.70223000	0.31383700

C	2.53133700	-1.44373000	-2.46731300
H	2.63027600	0.69231600	-2.09392400
C	2.43179700	-2.83595500	-0.49496800
H	2.46960900	-1.78289800	1.39676600
C	2.45736400	-2.70927500	-1.88412700
H	2.55971800	-1.34305100	-3.55008600
H	2.37575800	-3.82159100	-0.03867700
H	2.42135500	-3.59681400	-2.51197000
C	-2.46022200	-2.31583700	0.17651700
F	-2.83949000	-2.56245400	1.45144600
F	-1.39986400	-3.10246900	-0.07454300
F	-3.47424000	-2.78016400	-0.60135100

X=CH_3, TS1

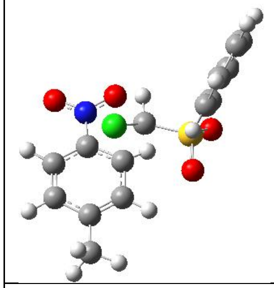

Thermochemistry:

Zero-point correction= 0.246872 (Hartree/Particle)
Thermal correction to Energy= 0.265982
Thermal correction to Enthalpy= 0.266927
Thermal correction to Gibbs Free Energy= 0.198374
Sum of electronic and zero-point Energies= -1753.988628
Sum of electronic and thermal Energies= -1753.969519
Sum of electronic and thermal Enthalpies= -1753.968574
Sum of electronic and thermal Free Energies= -1754.037127

Vibrational frequencies

**Frequency:**	**Infrared:**
**-163.14**	**472.6150**

Cartesian coordinates:

C	-2.84378900	0.96167800	0.01500300
C	-1.98090400	0.23607900	-0.84320500
C	-2.25196300	-1.15596700	-0.97393900
C	-3.17182400	-1.79940800	-0.17808200
C	-3.93934200	-1.04785300	0.75537100
C	-3.77902100	0.31406900	0.84287300
C	0.08159700	0.27507500	0.14665900
S	1.27337400	-0.35891000	-0.93290800
N	-2.73880600	2.37408800	0.07044500
O	-3.53109800	3.01100500	0.78312800
Cl	0.00115500	-0.54555500	1.70693200

O	1.19833900	0.48015800	-2.14484600
O	1.12983300	-1.82059600	-1.04480300
O	-1.85158000	2.92443300	-0.59444900
H	-1.46913400	0.76642700	-1.63590000
H	-4.36790700	0.91559500	1.52721200
H	-4.65868400	-1.55552600	1.39532900
H	0.21505000	1.34871400	0.27369200
H	-1.65137500	-1.72793500	-1.67667000
C	2.92765400	-0.10192800	-0.26822800
C	3.47333400	-1.04251300	0.60610700
C	3.61523100	1.07465100	-0.56812300
C	4.71851900	-0.79993500	1.18241400
H	2.92305400	-1.95582600	0.81173800
C	4.86211200	1.30587400	0.00662500
H	3.16915400	1.78403500	-1.26012800
C	5.41469300	0.37163600	0.88483200
H	5.14888500	-1.53117900	1.86344200
H	5.40688000	2.21646000	-0.23387600
H	6.38801900	0.55632900	1.33442000
C	-3.38626500	-3.28356400	-0.28497600
H	-3.12745700	-3.78311600	0.65795400
H	-4.43655400	-3.52260000	-0.49983000
H	-2.76605200	-3.72037900	-1.07406600

X=CH_3, TS2

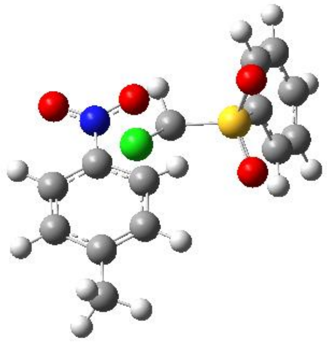

Thermochemistry:

Zero-point correction= 0.246552 (Hartree/Particle)
Thermal correction to Energy= 0.265777
Thermal correction to Enthalpy= 0.266721
Thermal correction to Gibbs Free Energy= 0.197911
Sum of electronic and zero-point Energies= -1753.980267
Sum of electronic and thermal Energies= -1753.961043
Sum of electronic and thermal Enthalpies= -1753.960098
Sum of electronic and thermal Free Energies= -1754.028909

Vibrational frequencies

Frequency: Infrared:

**-106.58**	**175.6181**		
**Cartesian coordinates:**			
C	1.82832000	1.54320900	-0.11705100
C	1.08191400	0.60318400	0.62526200
C	1.81560800	-0.37043100	1.34466000
C	3.18191700	-0.50179800	1.20542600
C	3.88867100	0.40902900	0.37668600
C	3.21900600	1.41909600	-0.27515100
C	-0.00320700	-0.83687600	-0.98122000
S	-1.42693900	-1.70426400	-0.45085000
N	1.15306700	2.62266300	-0.74727100
O	1.81687300	3.46437400	-1.37198800
Cl	1.26523600	-1.95516800	-1.51840600
O	-1.08121700	-2.56570500	0.69832300
O	-2.26737800	-2.30763300	-1.51201200
O	-0.07836700	2.68025200	-0.65222000
H	0.06362900	0.84373800	0.89316000
H	3.73814100	2.13726800	-0.90089900
H	4.96536200	0.30385800	0.25689600
H	-0.28290900	-0.17805700	-1.80714000
H	1.26463400	-1.07998700	1.95752100
C	-2.38532600	-0.32614200	0.19701700
C	-3.10591300	0.48753800	-0.67431100
C	-2.41152800	-0.10214100	1.57112700
C	-3.84430100	1.55098800	-0.16253800
H	-3.09096000	0.27861100	-1.74025600
C	-3.16341000	0.95604100	2.07770800
H	-1.85143400	-0.76753200	2.22266500
C	-3.87422700	1.78597000	1.21172900
H	-4.39584300	2.19983300	-0.83876700
H	-3.18924400	1.13609100	3.15021000
H	-4.45062700	2.61918100	1.60764000
C	3.93235300	-1.59458500	1.91387000
H	4.36588200	-2.29806900	1.19116200
H	4.76059200	-1.19124500	2.51161700
H	3.27478200	-2.16502200	2.57749400

X=CH$_3$, TS3

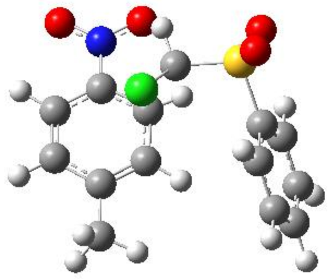

**Thermochemistry:**			
Zero-point correction= 0.246782 (Hartree/Particle)			
Thermal correction to Energy= 0.265823			
Thermal correction to Enthalpy= 0.266767			
Thermal correction to Gibbs Free Energy= 0.198725			
Sum of electronic and zero-point Energies= -1753.983101			
Sum of electronic and thermal Energies= -1753.964060			
Sum of electronic and thermal Enthalpies= -1753.963116			
Sum of electronic and thermal Free Energies= -1754.031158			
**Vibrational frequencies**			
**Frequency:**	**Infrared:**		
**-158.29**	**336.8935**		
**Cartesian coordinates:**			
C	2.58703700	0.01666700	-0.53567700
C	1.19508600	0.18764000	-0.74947200
C	0.67775500	1.48223700	-0.46473000
C	1.42274600	2.44204900	0.18528000
C	2.77938200	2.17474300	0.51052900
C	3.35282700	0.97940000	0.14240100
C	0.16806300	-1.13511500	0.83594900
S	-1.41897600	-1.77329500	0.48193800
N	3.21873300	-1.16919600	-0.99048600
O	4.44083700	-1.30528200	-0.81705200
Cl	0.22094400	-0.21968200	2.35073900
O	-2.24526900	-2.14281200	1.64862000
O	-1.23441200	-2.75313100	-0.60552400
O	2.52437200	-2.02738300	-1.54687000
H	0.70653100	-0.45217400	-1.47278900
H	4.39710600	0.76293900	0.34158200
H	3.36725700	2.92476800	1.03611600
H	0.86610700	-1.97303400	0.85765800
H	-0.35747000	1.69274600	-0.72212600
C	-2.27433000	-0.37197900	-0.25241100
C	-2.32890000	-0.26185700	-1.64022600
C	-2.86052500	0.59287500	0.56558000
C	-2.96858900	0.83403000	-2.21603400
H	-1.87664900	-1.04171400	-2.24689700
C	-3.49150600	1.68986700	-0.01749900
H	-2.82196000	0.47309200	1.64408300
C	-3.54472300	1.81341900	-1.40647500
H	-3.01548100	0.92441300	-3.29903600
H	-3.94672700	2.44912100	0.61477400
H	-4.03965000	2.67067800	-1.85792900
C	0.82538800	3.77466000	0.54270200
H	0.79570400	3.90770900	1.63224800
H	1.41818200	4.60305500	0.13228000

H	-0.19899300	3.86713800	0.16697000

X=CH_3, TS4

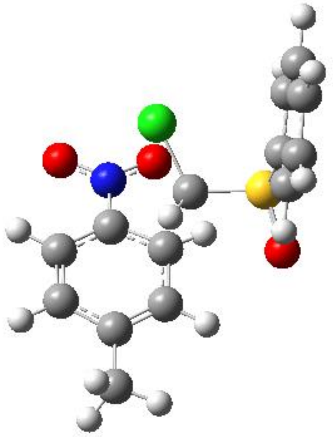

Thermochemistry:

Zero-point correction= 0.246914 (Hartree/Particle)
Thermal correction to Energy= 0.265980
Thermal correction to Enthalpy= 0.266924
Thermal correction to Gibbs Free Energy= 0.198630
Sum of electronic and zero-point Energies= -1753.986071
Sum of electronic and thermal Energies= -1753.967005
Sum of electronic and thermal Enthalpies= -1753.966061
Sum of electronic and thermal Free Energies= -1754.034355

Vibrational frequencies

Frequency:	Infrared:
**-165.79**	**500.2604**

Cartesian coordinates:

C	-2.89697900	-0.53127600	-0.06591300
C	-2.02249600	0.17611400	0.79417800
C	-2.13165500	1.59948000	0.77267900
C	-2.82127000	2.25451400	-0.22179300
C	-3.50790700	1.49493300	-1.21023900
C	-3.56359200	0.12142600	-1.11551600
C	0.02130200	-0.07120700	-0.14030100
S	1.28383200	0.09682100	1.03726400
N	-3.10318500	-1.92608200	0.12886900
O	-3.83198100	-2.53555600	-0.66860800
O	1.32134700	-1.07620100	1.92132500
O	1.10495300	1.44668900	1.61167300
O	-2.58047500	-2.47044300	1.10449700
H	-1.66440900	-0.32647200	1.68327400
H	-4.14149000	-0.47357100	-1.81488300
H	-4.03402600	2.00662900	-2.01432100
H	-1.57275700	2.16364900	1.51510200
C	2.89362800	0.12756000	0.22944500

C	3.44733700	1.34656200	-0.16231200
C	3.54247000	-1.07405600	-0.05699100
C	4.66399900	1.36226300	-0.83963800
H	2.92586900	2.26660300	0.08802300
C	4.75587700	-1.04994200	-0.74133100
H	3.09717300	-2.00828200	0.27189200
C	5.31880800	0.16485200	-1.13309600
H	5.10597000	2.31173700	-1.13477400
H	5.26644600	-1.98414400	-0.96510900
H	6.26842800	0.17925000	-1.66372700
Cl	0.08732200	-1.55575100	-1.08962300
H	0.02186800	0.79702200	-0.79960800
C	-2.87114100	3.75701600	-0.27616900
H	-2.44423100	4.13331300	-1.21572900
H	-3.90456700	4.12551300	-0.22387000
H	-2.30880200	4.20459700	0.54990700

X=CH_3, TS5

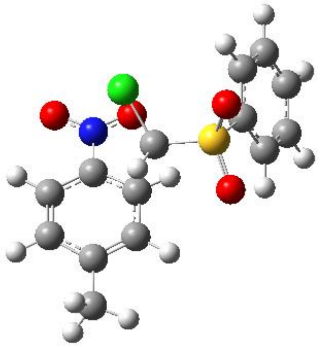

Thermochemistry:

Zero-point correction= 0.246646 (Hartree/Particle)
Thermal correction to Energy= 0.265790
Thermal correction to Enthalpy= 0.266734
Thermal correction to Gibbs Free Energy= 0.198213
Sum of electronic and zero-point Energies= -1753.983193
Sum of electronic and thermal Energies= -1753.964049
Sum of electronic and thermal Enthalpies= -1753.963105
Sum of electronic and thermal Free Energies= -1754.031626

Vibrational frequencies

**Frequency:**	**Infrared:**
**-94.43**	**192.2323**

Cartesian coordinates:

C	-2.01469400	1.24235300	-0.19119400
C	-1.28990500	0.19212700	-0.78836600
C	-2.03266500	-0.93577800	-1.22413300
C	-3.35656100	-1.10648600	-0.87695800
C	-4.00912000	-0.09714700	-0.12039500

C	-3.35006200	1.06977000	0.20291200
C	-0.13046300	-0.83757500	1.02979700
S	1.33321900	-1.68647400	0.63198300
N	-1.37822400	2.50084500	0.02679400
O	-2.02259100	3.40265100	0.57962400
O	0.92827900	-2.69705000	-0.37268400
O	2.18698200	-2.12248100	1.75743900
O	-0.22248300	2.65269100	-0.37470900
H	-0.31381400	0.40405600	-1.20192400
H	-3.84562400	1.87444000	0.73555800
H	-5.04805200	-0.23287800	0.17499100
H	-1.50827900	-1.71712700	-1.76942900
C	2.31930300	-0.46714800	-0.24482600
C	3.08450000	0.45127700	0.47152600
C	2.31170900	-0.45956500	-1.63744400
C	3.83642900	1.39744000	-0.21960100
H	3.08679700	0.41544900	1.55652200
C	3.07561300	0.48362300	-2.32168800
H	1.71927600	-1.20277600	-2.16397600
C	3.83353200	1.41554400	-1.61421500
H	4.42466000	2.12603900	0.33349900
H	3.07455100	0.49348600	-3.40949300
H	4.42037600	2.15876700	-2.14954300
Cl	0.01423700	0.28160100	2.39544700
H	-0.89498200	-1.58782500	1.23833400
C	-4.11701400	-2.33802200	-1.28526400
H	-4.47199200	-2.89196700	-0.40602700
H	-5.00241500	-2.08278100	-1.88286600
H	-3.49159000	-3.01538400	-1.87578500

X=Cl, TS1

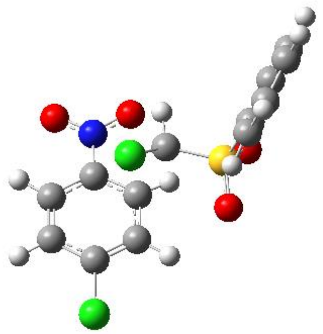

Thermochemistry:

Zero-point correction=	0.209868 (Hartree/Particle)
Thermal correction to Energy=	0.228611
Thermal correction to Enthalpy=	0.229555
Thermal correction to Gibbs Free Energy=	0.161054
Sum of electronic and zero-point Energies=	-2174.217365

Sum of electronic and thermal Energies=	-2174.198623		
Sum of electronic and thermal Enthalpies=	-2174.197679		
Sum of electronic and thermal Free Energies=	-2174.266179		
**Vibrational frequencies**			
**Frequency:**	**Infrared:**		
**-100.90**	**265.8025**		
**Cartesian coordinates:**			
C	-2.65169200	1.31724700	0.04076400
C	-1.82755200	0.54095100	-0.80452300
C	-2.15433800	-0.83353800	-0.92977300
C	-3.13065800	-1.37766500	-0.13263400
C	-3.88323500	-0.61245100	0.78432900
C	-3.63381200	0.73948000	0.85991200
C	0.30560000	0.41987900	0.24333600
S	1.42579800	-0.17841800	-0.92122500
N	-2.45853000	2.72764300	0.09507800
O	-3.20690000	3.40583900	0.81307300
Cl	0.16591400	-0.55198900	1.70848300
O	1.40928300	0.80055800	-2.02614700
O	1.15074100	-1.59882600	-1.19968300
O	-1.54632900	3.21787800	-0.57513200
H	-1.23630800	1.03278000	-1.56512500
H	-4.19495300	1.37954900	1.53191300
H	-4.63667100	-1.08535100	1.40517500
H	0.50631300	1.46476000	0.47555600
H	-1.57725300	-1.45722600	-1.60408900
C	3.10677300	-0.14777800	-0.27465800
C	3.58079800	-1.22735200	0.47143700
C	3.89229900	0.99104400	-0.45672800
C	4.85254800	-1.16226100	1.03754900
H	2.95587500	-2.10802500	0.58611700
C	5.16451500	1.04488900	0.10642500
H	3.50125100	1.81207700	-1.05174800
C	5.64590000	-0.02936000	0.85695400
H	5.22718000	-2.00251800	1.61830300
H	5.78461300	1.92614300	-0.04402700
H	6.63950800	0.01590900	1.29769800
Cl	-3.49708200	-3.08324700	-0.26653900

X=Cl, TS2

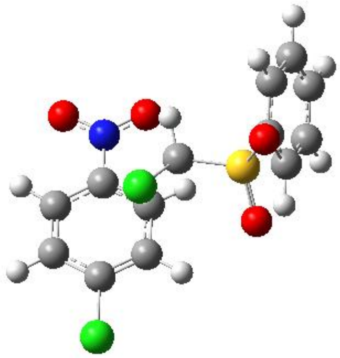

Thermochemistry:

Zero-point correction= 0.209552 (Hartree/Particle)
Thermal correction to Energy= 0.228396
Thermal correction to Enthalpy= 0.229340
Thermal correction to Gibbs Free Energy= 0.160602
Sum of electronic and zero-point Energies= -2174.208428
Sum of electronic and thermal Energies= -2174.189584
Sum of electronic and thermal Enthalpies= -2174.188640
Sum of electronic and thermal Free Energies= -2174.257377

Vibrational frequencies

Frequency: Infrared:
-49.40 77.1802

Cartesian coordinates:

C	1.50120600	1.83481000	-0.14604000
C	0.94288900	0.75409200	0.56016400
C	1.82350700	-0.22637700	1.05960900
C	3.16253400	-0.15695200	0.74658400
C	3.70736600	0.88737500	-0.02543800
C	2.86329500	1.88183900	-0.47264600
C	-0.25007700	-0.74339800	-1.10431300
S	-1.43030500	-1.78388700	-0.34939400
N	0.65092100	2.90067500	-0.57228200
O	1.15233700	3.85111900	-1.18717300
Cl	1.08108400	-1.68106500	-1.81546600
O	-0.83564200	-2.42047200	0.84606900
O	-2.22379500	-2.66406100	-1.23934900
O	-0.55095100	2.82662800	-0.31132600
H	-0.07257400	0.82340200	0.91846300
H	3.23412900	2.71321500	-1.06202500
H	4.76634300	0.89853900	-0.26097800
H	-0.74321200	-0.16167400	-1.88775100
H	1.42457500	-1.06438500	1.62173100
C	-2.57416200	-0.52615900	0.24186200
C	-3.49323300	0.04079400	-0.63838200
C	-2.54980600	-0.15914900	1.58434700
C	-4.37909300	1.00814100	-0.17132200

H	-3.51732100	-0.28779500	-1.67383000
C	-3.44710500	0.80078000	2.04800500
H	-1.83747900	-0.64248700	2.24777800
C	-4.35583100	1.38959900	1.17008000
H	-5.09121800	1.46423400	-0.85520200
H	-3.43247900	1.09345200	3.09550100
H	-5.04784300	2.14715200	1.53116400
Cl	4.24838500	-1.38977500	1.34138400

X=Cl, TS3

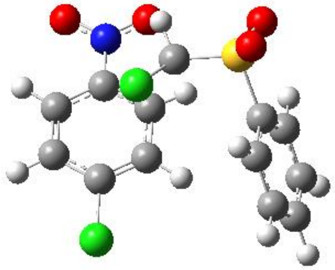

Thermochemistry:

Zero-point correction= 0.209756 (Hartree/Particle)
Thermal correction to Energy= 0.228457
Thermal correction to Enthalpy= 0.229401
Thermal correction to Gibbs Free Energy= 0.161307
Sum of electronic and zero-point Energies= -2174.212175
Sum of electronic and thermal Energies= -2174.193474
Sum of electronic and thermal Enthalpies= -2174.192530
Sum of electronic and thermal Free Energies= -2174.260624

Vibrational frequencies

Frequency:	Infrared:
**-81.22**	**161.1024**

Cartesian coordinates:

C	2.52200400	-0.47405400	-0.57508200
C	1.16965300	-0.13346900	-0.79857800
C	0.79860600	1.20738600	-0.54225000
C	1.69086600	2.05839500	0.06687300
C	3.00500400	1.67410800	0.39742800
C	3.41163800	0.39928800	0.06398700
C	-0.07330300	-1.29816700	0.93922500
S	-1.72294400	-1.74458300	0.61534800
N	2.99527500	-1.75488700	-0.98944900
O	4.18112600	-2.04633400	-0.78215100
Cl	0.11268900	-0.27262700	2.37015300
O	-2.60670600	-1.88501600	1.79017500
O	-1.66551600	-2.84037000	-0.37237900
O	2.20100200	-2.51825300	-1.54123000
H	0.57015000	-0.75756400	-1.44574100

H	4.42139300	0.05953300	0.26685600
H	3.67400400	2.37109300	0.89056600
H	0.51850000	-2.21017600	1.02479200
H	-0.20339500	1.54575100	-0.78417200
C	-2.36578300	-0.31481900	-0.26639000
C	-2.37898900	-0.32396700	-1.65992900
C	-2.82254300	0.79393900	0.44530000
C	-2.84259900	0.79546600	-2.34830200
H	-2.03447400	-1.21234600	-2.18252900
C	-3.27621100	1.91275700	-0.25026500
H	-2.82099400	0.76906100	1.53085000
C	-3.28491600	1.91684000	-1.64549600
H	-2.85645800	0.79318300	-3.43603500
H	-3.62348500	2.78470300	0.29935900
H	-3.63869100	2.79281100	-2.18477400
Cl	1.20459400	3.70198400	0.41731400

X=Cl, TS4

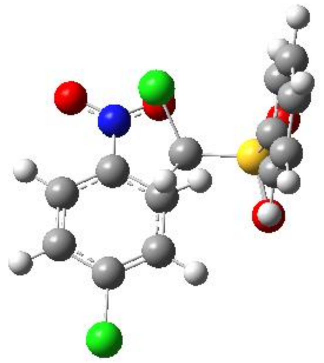

Thermochemistry:

Zero-point correction= 0.209889 (Hartree/Particle)
Thermal correction to Energy= 0.228620
Thermal correction to Enthalpy= 0.229564
Thermal correction to Gibbs Free Energy= 0.161148
Sum of electronic and zero-point Energies= -2174.215691
Sum of electronic and thermal Energies= -2174.196960
Sum of electronic and thermal Enthalpies= -2174.196016
Sum of electronic and thermal Free Energies= -2174.264432

Vibrational frequencies

**Frequency:**	**Infrared:**
**-85.85**	**245.7898**

Cartesian coordinates:

C	-2.73761100	-0.96070600	-0.03079600
C	-1.93216100	-0.19029800	0.83301700
C	-2.13809300	1.21359100	0.83103600
C	-2.91871100	1.77754500	-0.14746300

C	-3.58027000	1.00847300	-1.12836100
C	-3.49427500	-0.36666600	-1.04912700
C	0.22083300	-0.23130300	-0.18184600
S	1.44301500	0.00559900	1.01210700
N	-2.78480900	-2.38240700	0.12932700
O	-3.44722500	-3.04328500	-0.68006900
O	1.58241400	-1.19295900	1.85195200
O	1.12717100	1.30900800	1.63477700
O	-2.19676200	-2.88259700	1.08656800
H	-1.46180400	-0.67154300	1.67952500
H	-4.02870000	-1.00395800	-1.74518900
H	-4.17048500	1.49332500	-1.89888700
H	-1.60363800	1.82848600	1.54721800
C	3.05598600	0.22391900	0.23891900
C	3.49175500	1.50438600	-0.10305000
C	3.82187600	-0.89736700	-0.08229000
C	4.70631200	1.66260200	-0.76557300
H	2.88065900	2.35991700	0.17207400
C	5.03242000	-0.73093100	-0.75189000
H	3.46764800	-1.88193600	0.20842600
C	5.47704400	0.54611200	-1.09449300
H	5.05554400	2.66066800	-1.02214000
H	5.63334100	-1.60248600	-1.00261300
H	6.42454900	0.67218700	-1.61400500
Cl	0.38717600	-1.68170200	-1.16936900
H	0.13693100	0.65387200	-0.81206400
Cl	-3.12153100	3.51707000	-0.19331800

X=CN, TS1

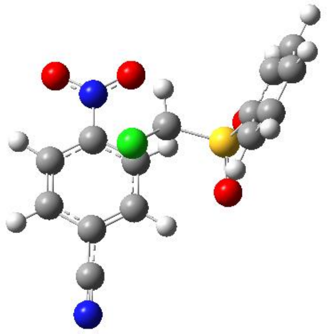

Thermochemistry:

Zero-point correction= 0.218117 (Hartree/Particle)
Thermal correction to Energy= 0.237284
Thermal correction to Enthalpy= 0.238228
Thermal correction to Gibbs Free Energy= 0.169210
Sum of electronic and zero-point Energies= -1806.910762
Sum of electronic and thermal Energies= -1806.891595
Sum of electronic and thermal Enthalpies= -1806.890650

Sum of electronic and thermal Free Energies= -1806.959669			
**Vibrational frequencies**			
**Frequency:**	**Infrared:**		
**-59.04**	**118.2723**		
**Cartesian coordinates:**			
C	2.83207200	-1.15960500	0.04745400
C	1.94414900	-0.46464200	-0.79657800
C	2.15183000	0.92648800	-0.93760400
C	3.09900600	1.57640400	-0.16489300
C	3.91911900	0.86382900	0.75231300
C	3.77791700	-0.49963100	0.84915400
C	-0.23520200	-0.38032700	0.29115500
S	-1.36958600	0.07786100	-0.92134200
N	2.74424800	-2.58424700	0.12413000
O	3.54991100	-3.18968000	0.84235200
Cl	-0.13867800	0.70287000	1.67695800
O	-1.31702300	-0.99230600	-1.93641700
O	-1.13433300	1.47670600	-1.32052300
O	1.86371000	-3.14804600	-0.52716500
H	1.35141700	-1.01215400	-1.51669500
H	4.39396400	-1.08740700	1.52047000
H	4.64463200	1.39756000	1.35758000
H	-0.38080900	-1.41477600	0.59835500
H	1.51194600	1.48882000	-1.61013600
C	-3.04938000	0.05437900	-0.27476800
C	-3.55907500	1.18483600	0.36427700
C	-3.79720300	-1.12127600	-0.34808500
C	-4.83034000	1.13377400	0.93286100
H	-2.96241700	2.09166000	0.39437600
C	-5.06908100	-1.16132400	0.21688300
H	-3.37836600	-1.98237700	-0.86204000
C	-5.58660400	-0.03588500	0.86072700
H	-5.23350000	2.01335100	1.43008600
H	-5.66062000	-2.07201200	0.15094900
H	-6.57993600	-0.07032100	1.30292400
C	3.28800900	2.98953700	-0.30219200
N	3.47791400	4.13286800	-0.40168300

**X=CN, TS2**

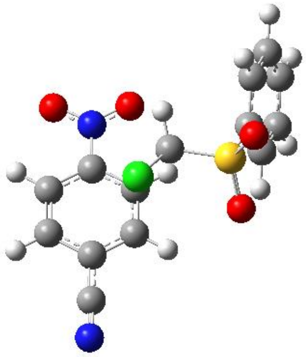

Thermochemistry:

Zero-point correction= 0.217821 (Hartree/Particle)
Thermal correction to Energy= 0.238002
Thermal correction to Enthalpy= 0.238947
Thermal correction to Gibbs Free Energy= 0.166394
Sum of electronic and zero-point Energies= -1806.901447
Sum of electronic and thermal Energies= -1806.881265
Sum of electronic and thermal Enthalpies= -1806.880321
Sum of electronic and thermal Free Energies= -1806.952874

Vibrational frequencies

**Frequency:**	**Infrared:**
-57.25	115.1386

Cartesian coordinates:

C	1.65676600	1.84578000	-0.10927600
C	1.04572000	0.78638200	0.57457400
C	1.86515500	-0.24870900	1.05054200
C	3.22450300	-0.24577700	0.76752500
C	3.81368200	0.80414900	0.01726100
C	3.02476000	1.84269000	-0.42155400
C	-0.19417600	-0.86250800	-1.08217700
S	-1.40207800	-1.81734000	-0.26405100
N	0.85430800	2.95851000	-0.52900600
O	1.40675900	3.89035500	-1.12401900
Cl	1.11845400	-1.87139600	-1.72198400
O	-0.82643100	-2.38408400	0.97494600
O	-2.21165400	-2.73708800	-1.09647800
O	-0.34995300	2.93352900	-0.27825400
H	0.01198000	0.86740100	0.87357000
H	3.43713400	2.66772600	-0.99134400
H	4.87573600	0.77810100	-0.20488300
H	-0.66619400	-0.31404000	-1.90146400
H	1.41231000	-1.07912900	1.58370000
C	-2.51524600	-0.49322300	0.23123300
C	-3.40532700	0.04472200	-0.69610000
C	-2.49660300	-0.04404700	1.54864100
C	-4.26412100	1.06809500	-0.30373400

H	-3.42914500	-0.34921900	-1.70854600
C	-3.36724800	0.97183200	1.93770300
H	-1.80980400	-0.50777000	2.25184400
C	-4.24408000	1.53356400	1.01094500
H	-4.95334700	1.50155000	-1.02475100
H	-3.35669000	1.32921500	2.96486300
H	-4.91458300	2.33480300	1.31326400
C	4.06337700	-1.29980700	1.25268500
N	4.77703400	-2.12679900	1.65231000

X=CN, TS3

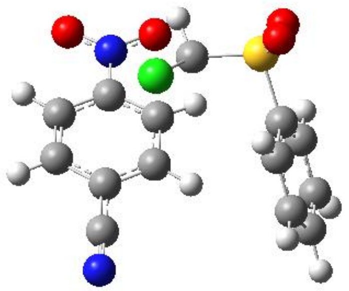

Thermochemistry:

Zero-point correction= 0.217966 (Hartree/Particle)
Thermal correction to Energy= 0.237123
Thermal correction to Enthalpy= 0.238067
Thermal correction to Gibbs Free Energy= 0.169347
Sum of electronic and zero-point Energies= -1806.905597
Sum of electronic and thermal Energies= -1806.886440
Sum of electronic and thermal Enthalpies= -1806.885496
Sum of electronic and thermal Free Energies= -1806.954216

Vibrational frequencies

**Frequency:**	**Infrared:**
**-25.38**	**52.0802**

Cartesian coordinates:

C	2.61856700	-0.29062000	-0.55656300
C	1.25535800	-0.03296600	-0.79002000
C	0.79211700	1.26738500	-0.51502300
C	1.61948000	2.19208300	0.10427100
C	2.96299600	1.87238000	0.43014500
C	3.45310900	0.63180200	0.09158100
C	-0.04524700	-1.21026600	1.02533400
S	-1.65685200	-1.74137500	0.65473400
N	3.17121100	-1.54529100	-0.97130800
O	4.36982600	-1.76091400	-0.75659300
Cl	0.05140900	-0.12973500	2.42085400
O	-2.57704200	-1.87901000	1.80067200
O	-1.50900100	-2.86869100	-0.28691700
O	2.42640800	-2.35191900	-1.52705100

H	0.67910100	-0.71598400	-1.39699900
H	4.48089700	0.35350500	0.29609700
H	3.58888600	2.60533700	0.92867200
H	0.59550600	-2.08371300	1.14799600
H	-0.23406500	1.52996900	-0.75069200
C	-2.32258100	-0.37399300	-0.30462500
C	-2.27760100	-0.43373700	-1.69646200
C	-2.84591200	0.74292800	0.34644600
C	-2.74778100	0.64433400	-2.44394700
H	-1.88247000	-1.32775200	-2.17158000
C	-3.30371000	1.82130000	-0.40802700
H	-2.89032400	0.75790900	1.43131600
C	-3.25308400	1.77546600	-1.80167800
H	-2.71663600	0.60255800	-3.53050100
H	-3.69888600	2.70116900	0.09418400
H	-3.60936000	2.62045900	-2.38654400
C	1.12562800	3.50407400	0.39647500
N	0.75071300	4.58061900	0.62837300

X=F, TS1

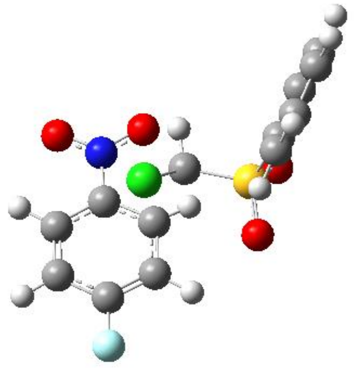

Thermochemistry:

Zero-point correction= 0.211215 (Hartree/Particle)
Thermal correction to Energy= 0.229553
Thermal correction to Enthalpy= 0.230497
Thermal correction to Gibbs Free Energy= 0.163270
Sum of electronic and zero-point Energies= -1813.931783
Sum of electronic and thermal Energies= -1813.913445
Sum of electronic and thermal Enthalpies= -1813.912501
Sum of electronic and thermal Free Energies= -1813.979728

Vibrational frequencies

**Frequency:**	**Infrared:**
**-111.21**	**306.4127**

Cartesian coordinates:

C	2.89695100	0.88810300	-0.04168100
C	2.01692200	0.20903000	0.83596000
C	2.23804000	-1.18035800	1.01131700

C	3.15903800	-1.81500100	0.22291400
C	3.96625800	-1.15849400	-0.72279400
C	3.82452300	0.20631000	-0.84469700
C	-0.10105700	0.25491200	-0.21432000
S	-1.28760400	-0.21382100	0.94441900
N	2.81574000	2.30524500	-0.14594600
O	3.60807100	2.89726500	-0.89327200
Cl	-0.03299100	-0.76059200	-1.65597000
O	-1.19760700	0.78232300	2.03028900
O	-1.14995400	-1.64749100	1.25510000
O	1.95173300	2.89079600	0.51232800
H	1.47889500	0.77585100	1.58349400
H	4.42863200	0.77756600	-1.54088800
H	4.66963300	-1.72448100	-1.32452800
H	-0.20837400	1.30693800	-0.47471500
H	1.62686100	-1.74792400	1.70507700
C	-2.94973300	-0.04093100	0.27066300
C	-3.51581000	-1.08985700	-0.45457000
C	-3.62391200	1.17263800	0.41074700
C	-4.76807500	-0.91865800	-1.04179100
H	-2.97644500	-2.02881800	-0.53661800
C	-4.87752100	1.33306500	-0.17340100
H	-3.16238600	1.96807900	0.98983900
C	-5.45069800	0.28969400	-0.90287800
H	-5.21443200	-1.73457900	-1.60638100
H	-5.41159000	2.27370800	-0.05610100
H	-6.42953900	0.41819800	-1.36002800
F	3.34039800	-3.15065700	0.36832200

X=F, TS2

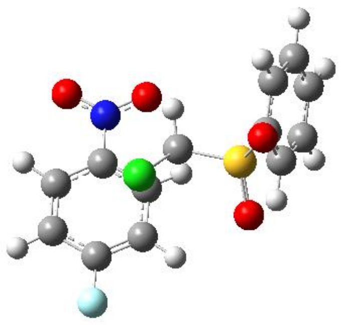

Thermochemistry:

Zero-point correction= 0.210912 (Hartree/Particle)
Thermal correction to Energy= 0.229348
Thermal correction to Enthalpy= 0.230292
Thermal correction to Gibbs Free Energy= 0.162871
Sum of electronic and zero-point Energies= -1813.922997
Sum of electronic and thermal Energies= -1813.904561

Sum of electronic and thermal Enthalpies= -1813.903617
Sum of electronic and thermal Free Energies= -1813.971038

Vibrational frequencies

Frequency: Infrared:
-65.75 110.0348

Cartesian coordinates:

C	1.92679700	1.50239100	-0.07609300
C	1.16036100	0.57651200	0.66152000
C	1.85122500	-0.45188100	1.33414600
C	3.20318900	-0.57984600	1.13983000
C	3.96264100	0.29414400	0.34685700
C	3.30648700	1.34083500	-0.26654500
C	-0.06683300	-0.84571000	-0.99316300
S	-1.41559000	-1.70433900	-0.29352100
N	1.27478000	2.61995800	-0.67682900
O	1.95469500	3.43293300	-1.31810200
Cl	1.18964900	-1.96707500	-1.56060900
O	-0.98506300	-2.36785900	0.95628100
O	-2.25605300	-2.50778600	-1.21253600
O	0.05491400	2.72893300	-0.53533000
H	0.14293000	0.82620000	0.91996000
H	3.83679700	2.05622400	-0.88525000
H	5.02844000	0.13016400	0.22636000
H	-0.42884100	-0.25107700	-1.83610400
H	1.30759100	-1.18204300	1.92464200
C	-2.42674100	-0.29393000	0.18567000
C	-3.20578500	0.35573800	-0.76945800
C	-2.44325100	0.11147200	1.51724700
C	-3.98923200	1.44177500	-0.38834700
H	-3.20352700	0.00001700	-1.79614300
C	-3.23904700	1.19134900	1.89447200
H	-1.84324500	-0.43539000	2.23973200
C	-4.00561800	1.86096200	0.94185700
H	-4.58991600	1.96176100	-1.13103100
H	-3.25608300	1.51367600	2.93321800
H	-4.61735900	2.71102600	1.23572600
F	3.86356300	-1.58591100	1.76032400

X=F, TS3

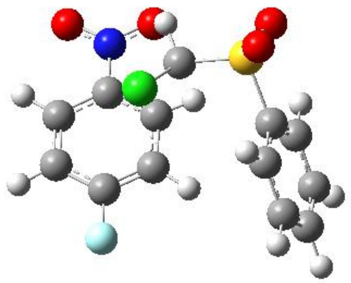

Thermochemistry:

Zero-point correction= 0.211122 (Hartree/Particle)
Thermal correction to Energy= 0.229404
Thermal correction to Enthalpy= 0.230348
Thermal correction to Gibbs Free Energy= 0.163592
Sum of electronic and zero-point Energies= -1813.926487
Sum of electronic and thermal Energies= -1813.908205
Sum of electronic and thermal Enthalpies= -1813.907260
Sum of electronic and thermal Free Energies= -1813.974017

Vibrational frequencies

**Frequency:**	**Infrared:**
**-98.85**	**203.3829**

Cartesian coordinates:

C	2.57527600	-0.01080500	-0.54453300
C	1.19528700	0.22980200	-0.75411700
C	0.71457500	1.51399600	-0.40493000
C	1.53635100	2.37409500	0.27558100
C	2.87422000	2.09109300	0.59145400
C	3.38754800	0.88343600	0.16589700
C	0.08842700	-1.17382300	0.85196000
S	-1.51856800	-1.73421000	0.48737300
N	3.15521200	-1.21151500	-1.04689200
O	4.36183700	-1.41821600	-0.85359800
Cl	0.18716700	-0.26684500	2.36968200
O	-2.37947200	-2.05343200	1.64404800
O	-1.37197000	-2.73020300	-0.59226200
O	2.42905100	-1.99803000	-1.65829800
H	0.66114700	-0.38428400	-1.46494200
H	4.42146200	0.61301100	0.35001400
H	3.46851200	2.81163300	1.14335500
H	0.75601500	-2.03614900	0.86185700
H	-0.30933700	1.80038300	-0.62115600
C	-2.29038600	-0.29525700	-0.26737200
C	-2.31055700	-0.18519500	-1.65650800
C	-2.83910800	0.70324300	0.53649500
C	-2.87557500	0.94356100	-2.24675400
H	-1.89126100	-0.99114000	-2.25287500
C	-3.39484700	1.83281900	-0.06093800

H	-2.82884000	0.58545200	1.61586400
C	-3.41201700	1.95623900	-1.45068700
H	-2.89504500	1.03378300	-3.33066000
H	-3.81595700	2.61956800	0.56098700
H	-3.84593600	2.84014000	-1.91310500
F	1.05525300	3.58797200	0.64043800

X=F, TS4

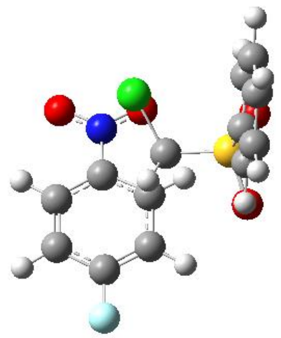

Thermochemistry:

Zero-point correction= 0.211252 (Hartree/Particle)
Thermal correction to Energy= 0.229564
Thermal correction to Enthalpy= 0.230508
Thermal correction to Gibbs Free Energy= 0.163439
Sum of electronic and zero-point Energies= -1813.929487
Sum of electronic and thermal Energies= -1813.911175
Sum of electronic and thermal Enthalpies= -1813.910231
Sum of electronic and thermal Free Energies= -1813.977300

Vibrational frequencies

Frequency:	Infrared:
**-107.39**	**324.9115**

Cartesian coordinates:

C	-2.93313800	-0.48349100	-0.03932200
C	-2.05443900	0.23324400	0.80620600
C	-2.14007100	1.65056300	0.77079600
C	-2.86554600	2.24193200	-0.22671600
C	-3.59700100	1.52456600	-1.18954200
C	-3.63605600	0.14905500	-1.07318700
C	0.05194700	-0.05842400	-0.18202300
S	1.29535600	0.08438200	1.00893400
N	-3.11084000	-1.88790800	0.15872700
O	-3.83399500	-2.50844800	-0.63165100
O	1.31886900	-1.10324800	1.87469800
O	1.10867800	1.42521900	1.60332700
O	-2.56798600	-2.41560800	1.12857400
H	-1.64899900	-0.26625300	1.67520400
H	-4.22839700	-0.45739000	-1.74952400
H	-4.13876700	2.05400800	-1.96658500

H	-1.55777300	2.24826500	1.46397300
C	2.92103700	0.12788800	0.23275700
C	3.47989600	1.35232500	-0.13421100
C	3.57580900	-1.06907400	-0.05939400
C	4.70700300	1.37793100	-0.79210500
H	2.95359100	2.26866500	0.11955900
C	4.79985300	-1.03513700	-0.72433000
H	3.12678800	-2.00797800	0.25068100
C	5.36764600	0.18510100	-1.09113200
H	5.15236900	2.33163000	-1.06784900
H	5.31493600	-1.96590600	-0.95216000
H	6.32552100	0.20739300	-1.60645000
Cl	0.09220000	-1.53582300	-1.14493300
H	0.06030400	0.81754500	-0.83083900
F	-2.90477200	3.59800700	-0.30139400

X=F, TS5

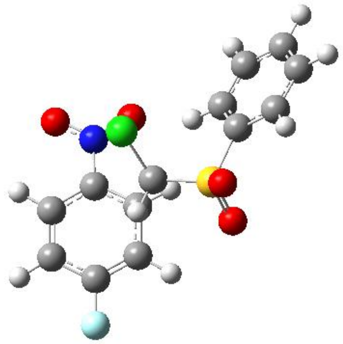

Thermochemistry:

Zero-point correction=	0.211373 (Hartree/Particle)
Thermal correction to Energy=	0.229605
Thermal correction to Enthalpy=	0.230549
Thermal correction to Gibbs Free Energy=	0.164007
Sum of electronic and zero-point Energies=	-1813.924821
Sum of electronic and thermal Energies=	-1813.906589
Sum of electronic and thermal Enthalpies=	-1813.905645
Sum of electronic and thermal Free Energies=	-1813.972187

Vibrational frequencies

**Frequency:**	**Infrared:**
**-93.13**	**158.5260**

Cartesian coordinates:

C	-2.33238400	1.15571500	-0.21332300
C	-1.60705400	0.13362300	-0.86594500
C	-2.29958300	-1.06902100	-1.15421600
C	-3.54120700	-1.26304200	-0.61026200
C	-4.20573400	-0.30539300	0.17469200
C	-3.59068400	0.91669800	0.35608300

C	-0.09114700	-0.75973500	0.78008300
S	1.29796500	-1.59623400	0.11807900
N	-1.76693800	2.45781700	-0.10342100
O	-2.41069300	3.34328300	0.47132700
O	0.83435500	-2.11146500	-1.19192100
O	1.98859400	-2.57155000	0.99294300
O	-0.65684000	2.65904300	-0.60778200
H	-0.72158600	0.37945400	-1.43189500
H	-4.07072800	1.71203200	0.91544500
H	-5.18238400	-0.52782800	0.59181400
H	-1.81056900	-1.84967300	-1.72716500
C	2.52316200	-0.31138300	-0.18593600
C	3.86512000	-0.67608200	-0.09581200
C	2.15466600	0.97667900	-0.56476600
C	4.85137700	0.25741300	-0.40500900
H	4.11604900	-1.68123100	0.23078600
C	3.14895500	1.90858800	-0.85847900
H	1.10937400	1.26739000	-0.59754900
C	4.49431300	1.55059000	-0.78748500
H	5.90042300	-0.02291600	-0.33694800
H	2.86176900	2.91977900	-1.13636000
H	5.26558900	2.28210800	-1.02011800
Cl	0.25844900	0.09643700	2.30683400
H	-0.81761400	-1.54908300	0.99123500
F	-4.18944000	-2.43075700	-0.84389100

X=H, TS1

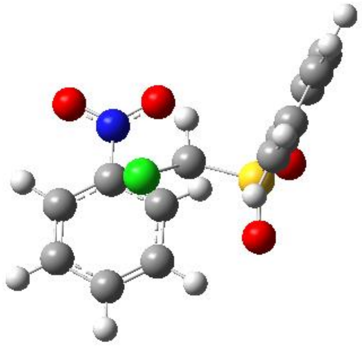

Thermochemistry:

Zero-point correction= 0.219431 (Hartree/Particle)
Thermal correction to Energy= 0.236900
Thermal correction to Enthalpy= 0.237844
Thermal correction to Gibbs Free Energy= 0.172693
Sum of electronic and zero-point Energies= -1714.736206
Sum of electronic and thermal Energies= -1714.718737
Sum of electronic and thermal Enthalpies= -1714.717793
Sum of electronic and thermal Free Energies= -1714.782944

Vibrational frequencies			
Frequency:	Infrared:		
-161.92	470.8677		
Cartesian coordinates:			
C	3.07453900	0.51091700	-0.03428700
C	2.14215600	-0.06897100	0.86345200
C	2.29701500	-1.46427500	1.10737700
C	3.18266400	-2.21898300	0.37679000
C	4.02321100	-1.62710300	-0.59797900
C	3.96760200	-0.26588100	-0.79462200
C	0.09666000	0.04475500	-0.15734300
S	-1.15307700	-0.30526600	0.98515800
N	3.08421800	1.92110300	-0.20036100
O	3.93438600	2.43519900	-0.94405900
Cl	0.08953400	-0.98952600	-1.58612600
O	-0.99233700	0.68617300	2.06625100
O	-1.15770600	-1.74292200	1.30552900
O	2.23511300	2.58893100	0.40239000
H	1.66687500	0.56677100	1.59937900
H	4.60833400	0.23637100	-1.51157600
H	4.70783200	-2.23923700	-1.17956200
H	0.07700900	1.09858200	-0.43232000
H	1.64509000	-1.93463500	1.83777800
C	-2.77249600	0.01974500	0.26720500
C	-3.41731400	-0.97867200	-0.46349300
C	-3.33109500	1.29350800	0.37988600
C	-4.63238800	-0.69601900	-1.08434000
H	-2.96675300	-1.96485300	-0.52374900
C	-4.54858300	1.56555700	-0.23826500
H	-2.81075400	2.04787600	0.96404200
C	-5.19997900	0.57333200	-0.97348100
H	-5.14005500	-1.47182100	-1.65365300
H	-4.99331900	2.55396200	-0.14317600
H	-6.15010800	0.78954900	-1.45731100
H	3.24587800	-3.29058900	0.55801200

X=H, TS2

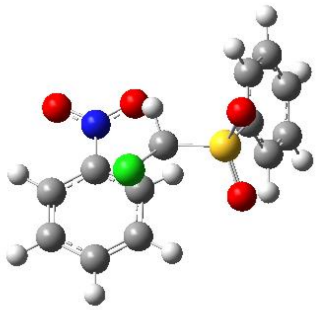

**Thermochemistry:**			
Zero-point correction= 0.219127 (Hartree/Particle)			
Thermal correction to Energy= 0.236698			
Thermal correction to Enthalpy= 0.237642			
Thermal correction to Gibbs Free Energy= 0.172269			
Sum of electronic and zero-point Energies= -1714.727690			
Sum of electronic and thermal Energies= -1714.710119			
Sum of electronic and thermal Enthalpies= -1714.709175			
Sum of electronic and thermal Free Energies= -1714.774548			
**Vibrational frequencies**			
**Frequency:**	**Infrared:**		
**-110.31**	**185.1449**		
**Cartesian coordinates:**			
C	2.19688300	1.19329000	0.04223000
C	1.29949400	0.40688100	0.79888900
C	1.87189800	-0.57336300	1.64665100
C	3.22288200	-0.83719600	1.60915900
C	4.08863500	-0.09803000	0.77057600
C	3.57301800	0.91463500	-0.00955400
C	0.15007300	-1.04630200	-0.74442500
S	-1.39833200	-1.66439200	-0.21281800
N	1.68455500	2.28010100	-0.71975000
O	2.47421700	2.99047500	-1.36037400
Cl	1.28501400	-2.36841900	-1.07473900
O	-1.23591900	-2.35011800	1.08588800
O	-2.22169300	-2.35440300	-1.23349400
O	0.46364700	2.47274000	-0.71778000
H	0.30201500	0.78292500	0.97178800
H	4.20189600	1.51849000	-0.65501600
H	5.15112300	-0.32585600	0.74043800
H	-0.00015200	-0.45791800	-1.65310900
H	1.21097400	-1.16670600	2.27235400
C	-2.23818500	-0.10785800	0.11667500
C	-2.79824900	0.61171200	-0.93634700
C	-2.34196000	0.34566100	1.42873800
C	-3.45070300	1.81257500	-0.67159600
H	-2.72883000	0.22327200	-1.94855800
C	-3.00699400	1.54245800	1.68796100
H	-1.91089300	-0.25270900	2.22689800
C	-3.55522600	2.27859400	0.63863000
H	-3.87723100	2.38729700	-1.49032200
H	-3.09182000	1.90337400	2.71058300
H	-4.06340800	3.21874500	0.84125900
H	3.63110800	-1.62612000	2.23813500

X=H, TS3

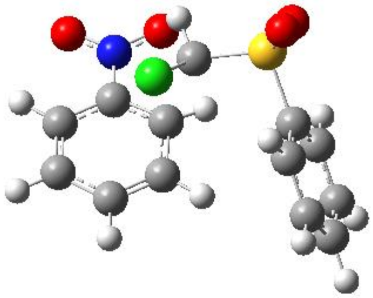

Thermochemistry:

Zero-point correction= 0.219364 (Hartree/Particle)
Thermal correction to Energy= 0.236755
Thermal correction to Enthalpy= 0.237699
Thermal correction to Gibbs Free Energy= 0.173071
Sum of electronic and zero-point Energies= -1714.730591
Sum of electronic and thermal Energies= -1714.713200
Sum of electronic and thermal Enthalpies= -1714.712255
Sum of electronic and thermal Free Energies= -1714.776884

Vibrational frequencies

**Frequency:**	**Infrared:**
**-159.45**	**346.6249**

Cartesian coordinates:

C	2.61052700	0.39719100	-0.43499500
C	1.21058800	0.54747700	-0.61968200
C	0.65009200	1.76002700	-0.12715700
C	1.38390500	2.61408500	0.66289500
C	2.74512100	2.35994300	0.95101800
C	3.35300800	1.25692400	0.39108500
C	0.23370200	-1.05332100	0.72198200
S	-1.32264300	-1.68940700	0.24653800
N	3.28010800	-0.67420400	-1.08513400
O	4.50663700	-0.79539300	-0.93785400
Cl	0.24074600	-0.40075200	2.36654000
O	-2.14472900	-2.26459000	1.32978200
O	-1.08700000	-2.48222300	-0.97509200
O	2.61131300	-1.44831700	-1.77831500
H	0.74536400	0.01669200	-1.44005300
H	4.40450200	1.04126700	0.54833900
H	3.30910100	3.03407800	1.59042000
H	0.96750600	-1.85273000	0.61159100
H	-0.39187800	1.97929600	-0.34274300
C	-2.22508300	-0.22525100	-0.27854900
C	-2.26670500	0.09782200	-1.63317700
C	-2.86008300	0.57712300	0.66850800
C	-2.94302300	1.24511100	-2.04299500
H	-1.77606500	-0.55971800	-2.34569300
C	-3.52798200	1.72659200	0.25163700

H	-2.82924300	0.29211200	1.71583700
C	-3.56874300	2.06330200	-1.10196500
H	-2.97978500	1.50195800	-3.09937200
H	-4.02090800	2.36001100	0.98590500
H	-4.09219200	2.96106600	-1.42382200
H	0.91074800	3.51073600	1.05993900

X=H, TS4

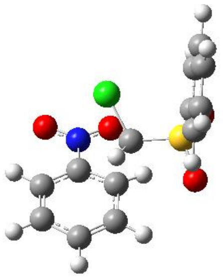

Thermochemistry:

Zero-point correction= 0.219464 (Hartree/Particle)
Thermal correction to Energy= 0.236899
Thermal correction to Enthalpy= 0.237843
Thermal correction to Gibbs Free Energy= 0.172881
Sum of electronic and zero-point Energies= -1714.734021
Sum of electronic and thermal Energies= -1714.716586
Sum of electronic and thermal Enthalpies= -1714.715642
Sum of electronic and thermal Free Energies= -1714.780604

Vibrational frequencies

Frequency:	Infrared:
**-167.13**	**506.5703**

Cartesian coordinates:

C	-3.05575300	-0.05101800	-0.09245700
C	-2.12104900	0.61662000	0.73782200
C	-2.09377800	2.04068700	0.63239400
C	-2.72148200	2.67828400	-0.41041300
C	-3.47541500	1.94880100	-1.36340400
C	-3.65722600	0.59241200	-1.18616400
C	-0.10428200	0.12558700	-0.15425100
S	1.16081500	0.21223800	1.02968900
N	-3.39241800	-1.40888900	0.18254500
O	-4.17372800	-1.99321000	-0.58254000
O	1.09049800	-0.93520600	1.94450500
O	1.09781800	1.58762800	1.56623300
O	-2.92341800	-1.94053700	1.19134500
H	-1.81993200	0.13243200	1.65760200
H	-4.28548200	0.00892300	-1.85077000
H	-3.94742300	2.45972800	-2.19907100
H	-1.49036700	2.59668900	1.34415800

C	2.76949600	0.08064900	0.23036400
C	3.42192800	1.23492300	-0.20350500
C	3.31627200	-1.18004200	-0.01212300
C	4.63420700	1.12571200	-0.87980900
H	2.97873300	2.20341000	0.01264100
C	4.52610500	-1.28077900	-0.69578500
H	2.79557800	-2.06201100	0.34891700
C	5.18684400	-0.13157600	-1.13025400
H	5.15256300	2.02423400	-1.20826100
H	4.95708300	-2.26145800	-0.88567300
H	6.13306000	-0.21489000	-1.66060400
H	-2.64599500	3.76082300	-0.50249500
Cl	-0.18392800	-1.39382600	-1.04432500
H	-0.01172200	0.96260400	-0.84657800

X=H, TS5

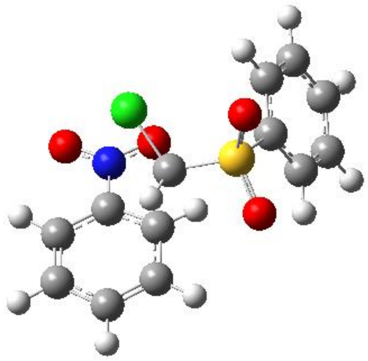

Thermochemistry:

Zero-point correction= 0.219214 (Hartree/Particle)
Thermal correction to Energy= 0.236711
Thermal correction to Enthalpy= 0.237655
Thermal correction to Gibbs Free Energy= 0.172575
Sum of electronic and zero-point Energies= -1714.731112
Sum of electronic and thermal Energies= -1714.713616
Sum of electronic and thermal Enthalpies= -1714.712672
Sum of electronic and thermal Free Energies= -1714.777752

Vibrational frequencies

**Frequency:**	**Infrared:**
**-94.90**	**190.9596**

Cartesian coordinates:

C	-2.34233500	0.78633000	-0.23196400
C	-1.42823900	0.01196500	-0.97627700
C	-1.96518800	-1.07293200	-1.71795900
C	-3.26904900	-1.47408500	-1.53096900
C	-4.12136900	-0.77733900	-0.64301600
C	-3.65984900	0.35895600	-0.00896600
C	-0.22721000	-1.23866900	0.66442300

S	1.40061500	-1.70388900	0.26662300
N	-1.91442100	2.03602400	0.31418000
O	-2.71647000	2.69253600	0.99123200
O	1.27654600	-2.50958600	-0.96986100
O	2.23526100	-2.23229500	1.36625700
O	-0.77365600	2.42731100	0.05972200
H	-0.47464000	0.44759500	-1.23925900
H	-4.29683300	0.95056000	0.63976700
H	-5.14326800	-1.11334100	-0.48555700
H	-1.30362600	-1.63040100	-2.37557300
C	2.19021300	-0.16233400	-0.21043900
C	2.69876300	0.68917100	0.76860700
C	2.28651900	0.16299400	-1.56118500
C	3.29263300	1.88885200	0.38636400
H	2.62723300	0.40685100	1.81442000
C	2.89306500	1.36023000	-1.93587100
H	1.89734200	-0.53352000	-2.29881000
C	3.39023300	2.22630100	-0.96332800
H	3.67725400	2.56515900	1.14624300
H	2.97167700	1.61939800	-2.98949100
H	3.85162500	3.16672100	-1.25667900
H	-3.65023700	-2.33692200	-2.07451300
Cl	-0.40721900	-0.46918600	2.24884400
H	-0.84527200	-2.13625000	0.60856500

<u>Addition into *para* position relative to the nitro group:</u>

X=H, TS1

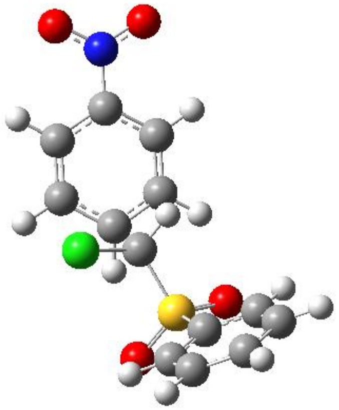

Thermochemistry:

Zero-point correction=	0.219761 (Hartree/Particle)
Thermal correction to Energy=	0.237160
Thermal correction to Enthalpy=	0.238104
Thermal correction to Gibbs Free Energy=	0.172920
Sum of electronic and zero-point Energies=	-1714.740186
Sum of electronic and thermal Energies=	-1714.722787

Sum of electronic and thermal Enthalpies=	-1714.721843
Sum of electronic and thermal Free Energies=	-1714.787027

Vibrational frequencies

**Frequency:**	**Infrared:**
-179.26	817.9101

Cartesian coordinates:

C	2.08297100	0.56678300	1.50764200
C	1.58386900	-0.74611900	1.24194300
C	2.38306400	-1.59393700	0.41821000
C	3.46467500	-1.09535300	-0.27131400
C	3.85123700	0.24614400	-0.09864500
C	3.16378500	1.06473600	0.82232700
C	-0.24437900	-0.36060200	0.00099000
S	-1.61243700	-0.20715200	1.05703400
Cl	-0.43119400	-1.59725400	-1.24432600
O	-1.29892200	0.93039100	1.94372000
O	-1.93432100	-1.52111000	1.63737500
H	0.92640300	-1.20798700	1.96970500
H	3.52181800	2.07513600	0.99069500
H	0.00040800	0.60083600	-0.44977700
H	2.10204600	-2.63623400	0.29798600
C	-3.08008500	0.27932000	0.13761400
C	-3.87810400	-0.69936900	-0.45544400
C	-3.36468000	1.63394900	-0.03645400
C	-4.97077000	-0.31374300	-1.22900600
H	-3.64163500	-1.74658700	-0.29250100
C	-4.46225900	2.01016100	-0.80603600
H	-2.73292600	2.37344400	0.44830200
C	-5.26476700	1.03826300	-1.40605800
H	-5.59764600	-1.07269600	-1.69199200
H	-4.69515900	3.06498400	-0.93478500
H	-6.12022600	1.33509400	-2.00886700
H	4.05076100	-1.72593300	-0.93190200
N	4.97557800	0.75282400	-0.79413100
O	5.58870000	0.00439100	-1.57056000
O	5.31313800	1.93181500	-0.60676100
H	1.55030900	1.19262300	2.21822900

X=H, TS2

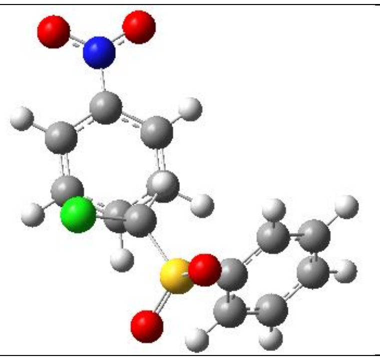

Thermochemistry:

Zero-point correction= 0.219600 (Hartree/Particle)
Thermal correction to Energy= 0.237029
Thermal correction to Enthalpy= 0.237973
Thermal correction to Gibbs Free Energy= 0.172924
Sum of electronic and zero-point Energies= -1714.731448
Sum of electronic and thermal Energies= -1714.714019
Sum of electronic and thermal Enthalpies= -1714.713075
Sum of electronic and thermal Free Energies= -1714.778124

Vibrational frequencies

**Frequency:**	**Infrared:**
-130.58	391.9158

Cartesian coordinates:

C	0.97867800	1.06551300	0.61782500
C	0.71730000	-0.20138300	1.21383200
C	1.81990400	-1.08136200	1.39548500
C	3.04649200	-0.80492900	0.83282800
C	3.24295800	0.40095900	0.13792500
C	2.20238700	1.34657900	0.05626800
C	-0.56783900	-1.33990000	-0.37468300
S	-2.31036700	-1.19021300	-0.26664700
Cl	-0.06957900	-3.03901000	-0.35063900
O	-2.77653800	-1.76188700	1.01110500
O	-3.07453200	-1.53533900	-1.48665900
H	-0.15167700	-0.31900900	1.85016700
H	2.39591600	2.29739500	-0.42921700
H	-0.23266400	-0.87710700	-1.30676400
H	1.67736000	-2.00615900	1.94610800
C	-2.43066800	0.59783500	-0.11303800
C	-2.30595200	1.40111900	-1.24512300
C	-2.66278500	1.15789300	1.14039600
C	-2.38836200	2.78540700	-1.11312600
H	-2.15970900	0.93973700	-2.21793900
C	-2.75630600	2.54273500	1.26354400
H	-2.77490300	0.49886100	1.99701400
C	-2.61125300	3.35681400	0.13997000
H	-2.28265600	3.41909600	-1.99058700

H	-2.93797000	2.98798700	2.23909400
H	-2.67586100	4.43797800	0.23992000
H	3.87998000	-1.49213900	0.93375800
N	4.51242500	0.69671100	-0.42298100
O	4.66713300	1.77670700	-1.01150900
O	5.42546000	-0.13281400	-0.30748000
H	0.18441600	1.80593900	0.57026100

X=H, TS3

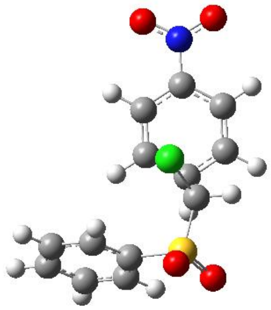

Thermochemistry:

Zero-point correction= 0.219556 (Hartree/Particle)
Thermal correction to Energy= 0.236953
Thermal correction to Enthalpy= 0.237897
Thermal correction to Gibbs Free Energy= 0.172943
Sum of electronic and zero-point Energies= -1714.735336
Sum of electronic and thermal Energies= -1714.717939
Sum of electronic and thermal Enthalpies= -1714.716995
Sum of electronic and thermal Free Energies= -1714.781949

Vibrational frequencies

**Frequency:**	**Infrared:**
-142.12	474.3238

Cartesian coordinates:

C	1.82923700	-0.97164300	-1.64902500
C	0.73097600	-0.15320500	-1.25570500
C	1.01811600	0.98569700	-0.45501100
C	2.27672200	1.18989100	0.06360500
C	3.32315100	0.30757800	-0.25992100
C	3.08850500	-0.77107500	-1.13314600
C	-0.48935200	-1.54255100	0.13540100
S	-2.21421800	-1.46239900	-0.08655900
Cl	0.04250700	-1.27951100	1.79542300
O	-3.05636100	-2.02752800	0.98639300
O	-2.44185700	-1.90768600	-1.47918700
H	-0.16483300	-0.16331500	-1.86559400
H	3.92031000	-1.41108100	-1.40859100
H	-0.14380500	-2.50658100	-0.23992200
H	0.21759200	1.67977200	-0.21219900

C	-2.55269300	0.30254100	-0.05762100
C	-2.71453800	0.98511400	-1.26127100
C	-2.64436200	0.97239500	1.16215800
C	-2.95728400	2.35734800	-1.24406500
H	-2.66098400	0.42919300	-2.19318800
C	-2.87767100	2.34550100	1.17035200
H	-2.53768900	0.41673800	2.08860500
C	-3.03231300	3.03962400	-0.03020100
H	-3.08600500	2.89454900	-2.18105000
H	-2.94178200	2.87499400	2.11817900
H	-3.21534200	4.11182500	-0.01888900
H	2.48889300	2.03885600	0.70495000
N	4.62481600	0.53269700	0.25226900
O	4.81498500	1.50564300	0.99659200
O	5.53410800	-0.24987300	-0.06205300
H	1.65685900	-1.79148000	-2.34268000

X=H, TS4

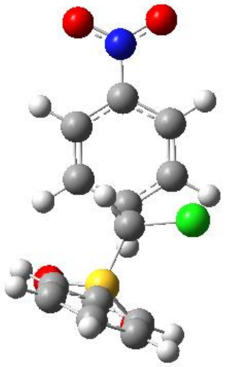

Thermochemistry:

Zero-point correction= 0.219760 (Hartree/Particle)
Thermal correction to Energy= 0.237160
Thermal correction to Enthalpy= 0.238104
Thermal correction to Gibbs Free Energy= 0.172916
Sum of electronic and zero-point Energies= -1714.740185
Sum of electronic and thermal Energies= -1714.722786
Sum of electronic and thermal Enthalpies= -1714.721842
Sum of electronic and thermal Free Energies= -1714.787029

Vibrational frequencies

**Frequency:**	**Infrared:**
-179.26	817.8189

Cartesian coordinates:

C	-2.38218600	-1.59080800	0.42633000
C	-1.58336400	-0.73863300	1.24591400
C	-2.08329100	0.57517600	1.50561600
C	-3.16457700	1.06921800	0.81821800
C	-3.85167100	0.24588500	-0.09876800

C	-3.46427000	-1.09616400	-0.26526000
C	0.24417800	-0.35740000	0.00274800
S	1.61357400	-0.20644000	1.05745600
O	1.93388700	-1.52102800	1.63722000
O	1.30273800	0.93141400	1.94469600
H	-0.92554300	-1.19663300	1.97578400
H	-4.05003200	-1.73018400	-0.92283700
H	-1.55105300	1.20455000	2.21341000
C	3.08112400	0.27785800	0.13674700
C	3.36791600	1.63204800	-0.03713700
C	3.87689800	-0.70201700	-0.45736900
C	4.46545200	2.00660000	-0.80759600
H	2.73792100	2.37249300	0.44845700
C	4.96952000	-0.31803700	-1.23181200
H	3.63876900	-1.74888200	-0.29456900
C	5.26571400	1.03351500	-1.40868300
H	4.70008000	3.06106000	-0.93618400
H	5.59464200	-1.07792800	-1.69563000
H	6.12113700	1.32906400	-2.01217300
H	-3.52335800	2.08010200	0.98201800
N	-4.97643000	0.74865100	-0.79640200
O	-5.31457400	1.92835700	-0.61458200
O	-5.58933600	-0.00378800	-1.56911000
H	-2.10047700	-2.63345500	0.31086800
Cl	0.42732200	-1.59442800	-1.24266100
H	0.00086700	0.60449200	-0.44786300

X=H, TS5

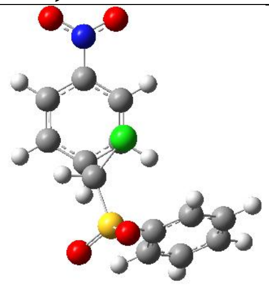

Thermochemistry:

Zero-point correction= 0.219556 (Hartree/Particle)
Thermal correction to Energy= 0.236953
Thermal correction to Enthalpy= 0.237897
Thermal correction to Gibbs Free Energy= 0.172945
Sum of electronic and zero-point Energies= -1714.735336
Sum of electronic and thermal Energies= -1714.717940
Sum of electronic and thermal Enthalpies= -1714.716995
Sum of electronic and thermal Free Energies= -1714.781947

Vibrational frequencies

Frequency:	Infrared:		
-142.15	474.3190		
**Cartesian coordinates:**			
C	-1.01769000	0.98517700	-0.45577000
C	-0.73079300	-0.15405000	-1.25609500
C	-1.82919500	-0.97258200	-1.64883300
C	-3.08833900	-0.77172800	-1.13276100
C	-3.32273300	0.30729100	-0.25992500
C	-2.27616800	1.18965200	0.06304100
C	0.48984400	-1.54301700	0.13513000
S	2.21471600	-1.46209100	-0.08649200
O	2.44286800	-1.90734000	-1.47904700
O	3.05687400	-2.02676000	0.98668800
H	0.16488200	-0.16444400	-1.86618400
H	-2.48814200	2.03888000	0.70410400
H	-1.65702700	-1.79271300	-2.34219400
C	2.55229700	0.30300700	-0.05753000
C	2.64352200	0.97286600	1.16227600
C	2.71376600	0.98571900	-1.26115200
C	2.87602000	2.34611000	1.17052900
H	2.53711100	0.41710600	2.08869300
C	2.95570700	2.35809400	-1.24388700
H	2.66057800	0.42980400	-2.19309300
C	3.03029300	3.04036800	-0.02999300
H	2.93977500	2.87561000	2.11837600
H	3.08413700	2.89540600	-2.18084800
H	3.21267900	4.11267700	-0.01863700
H	-3.92024100	-1.41179900	-1.40776100
N	-4.62427100	0.53270000	0.25245300
O	-4.81421700	1.50596000	0.99642500
O	-5.53368300	-0.24993500	-0.06136000
H	-0.21706300	1.67929400	-0.21341500
Cl	-0.04240900	-1.28029100	1.79507000
H	0.14477900	-2.50716100	-0.24035600

X=H, TS6

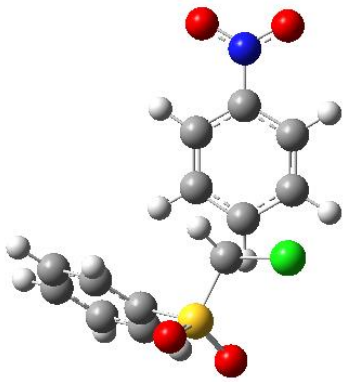

Thermochemistry:

Zero-point correction= 0.219600 (Hartree/Particle)
Thermal correction to Energy= 0.237029
Thermal correction to Enthalpy= 0.237973
Thermal correction to Gibbs Free Energy= 0.172923
Sum of electronic and zero-point Energies= -1714.731449
Sum of electronic and thermal Energies= -1714.714021
Sum of electronic and thermal Enthalpies= -1714.713076
Sum of electronic and thermal Free Energies= -1714.778126

Vibrational frequencies

**Frequency:**	**Infrared:**
-130.24	390.8479

Cartesian coordinates:

C	-1.81996600	-1.08134900	1.39570200
C	-0.71748600	-0.20123200	1.21412000
C	-0.97888600	1.06553200	0.61789500
C	-2.20258900	1.34642500	0.05621100
C	-3.24306600	0.40071900	0.13791200
C	-3.04654500	-0.80509300	0.83291900
C	0.56819700	-1.34000500	-0.37437900
S	2.31070500	-1.19002500	-0.26688800
O	3.07462100	-1.53482200	-1.48715600
O	2.77740300	-1.76176800	1.01064500
H	0.15158400	-0.31882700	1.85032500
H	-3.87996400	-1.49239200	0.93380900
H	-0.18467400	1.80600800	0.57029000
C	2.43061100	0.59803800	-0.11309900
C	2.66272500	1.15802000	1.14037200
C	2.30555400	1.40141700	-1.24507900
C	2.75589300	2.54287000	1.26366500
H	2.77511800	0.49891800	1.99690200
C	2.38761300	2.78571400	-1.11293600
H	2.15932500	0.94009800	-2.21792800
C	2.61049400	3.35704000	0.14019700
H	2.93755500	2.98806300	2.23924300

H	2.28164200	3.41947400	-1.99031400
H	2.67482900	4.43820900	0.24026200
H	-2.39616800	2.29715800	-0.42941600
N	-4.51253700	0.69629800	-0.42313400
O	-4.66729700	1.77622000	-1.01176600
O	-5.42548800	-0.13330800	-0.30762200
H	-1.67735200	-2.00609900	1.94638500
Cl	0.07016300	-3.03919400	-0.35029100
H	0.23261800	-0.87715600	-1.30628600

X=F, TS1

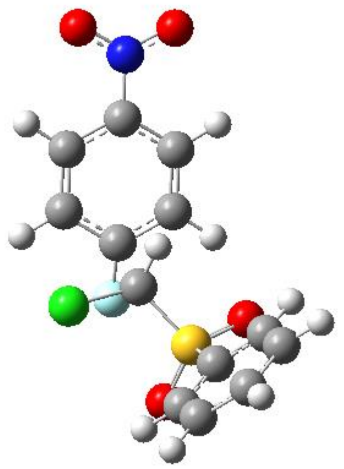

Thermochemistry:

Zero-point correction= 0.211171 (Hartree/Particle)
Thermal correction to Energy= 0.229523
Thermal correction to Enthalpy= 0.230467
Thermal correction to Gibbs Free Energy= 0.163149
Sum of electronic and zero-point Energies= -1813.931039
Sum of electronic and thermal Energies= -1813.912687
Sum of electronic and thermal Enthalpies= -1813.911743
Sum of electronic and thermal Free Energies= -1813.979061

Vibrational frequencies

**Frequency:**	**Infrared:**
-82.99	312.6068

Cartesian coordinates:

C	-2.11944300	-0.55191600	-1.48437400
C	-1.80513800	-1.47135000	-0.46483500
C	-2.51159900	-1.46303600	0.75197500
C	-3.41017000	-0.44836500	1.00502900
C	-3.65904300	0.52162100	0.02372400
C	-3.01946800	0.45722100	-1.22651100
C	0.30691600	-0.49452600	0.32763000
S	1.62206900	-0.68792300	-0.77286600
Cl	0.64604400	-1.05766600	1.97696700

O	1.15140400	-0.11331600	-2.05091700
O	2.14386600	-2.06210100	-0.72888400
H	-3.24967100	1.20624100	-1.97612200
H	-0.00336000	0.55100900	0.35172700
H	-2.29143000	-2.22536600	1.49174100
C	3.01724700	0.34691200	-0.28172600
C	3.94574600	-0.14087300	0.63833400
C	3.11624400	1.65164900	-0.76557900
C	4.97809900	0.68679800	1.07554200
H	3.85531200	-1.16505100	0.98789600
C	4.15474700	2.47071900	-0.32946200
H	2.38719200	2.00032800	-1.49228900
C	5.08545600	1.99155700	0.59397400
H	5.70517200	0.30940000	1.79163100
H	4.24115800	3.48479800	-0.71434600
H	5.89487200	2.63360800	0.93496800
H	-3.94057800	-0.39235100	1.94932000
N	-4.60360100	1.56022400	0.28309700
O	-5.17001300	1.58475600	1.38077700
O	-4.82019900	2.39524700	-0.60075900
H	-1.58321300	-0.61391600	-2.42501800
F	-1.18268000	-2.61092700	-0.81198100

X=F, TS2

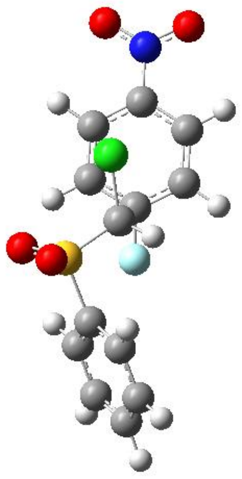

Thermochemistry:

Zero-point correction= 0.210724 (Hartree/Particle)
Thermal correction to Energy= 0.229212
Thermal correction to Enthalpy= 0.230156
Thermal correction to Gibbs Free Energy= 0.162695
Sum of electronic and zero-point Energies= -1813.920797
Sum of electronic and thermal Energies= -1813.902309
Sum of electronic and thermal Enthalpies= -1813.901365
Sum of electronic and thermal Free Energies= -1813.968825

Vibrational frequencies			
**Frequency:**	**Infrared:**		
-99.37	252.1852		
**Cartesian coordinates:**			
C	-1.63812400	-1.54549700	-1.03799600
C	-0.87903400	-1.24374300	0.10357200
C	-1.47148800	-0.64926000	1.22712600
C	-2.80131500	-0.28791200	1.17375700
C	-3.55931600	-0.57000700	0.02729500
C	-2.96950800	-1.18594700	-1.08370100
C	0.44453400	0.79277000	-0.68623900
S	1.70374900	1.36898500	0.38723900
Cl	-0.85589500	2.00629800	-0.82341900
O	1.27531100	1.22160000	1.79497800
O	2.34730800	2.65655800	0.03201100
H	-3.57612500	-1.39038600	-1.95904200
H	0.89756100	0.66532800	-1.67607600
H	-0.85197000	-0.41748900	2.08706200
C	2.96073500	0.11041200	0.08516100
C	3.82222500	0.25480700	-1.00024500
C	3.09121600	-0.96015300	0.96339700
C	4.81268200	-0.70042900	-1.22094900
H	3.72363400	1.12321500	-1.64619700
C	4.08984700	-1.90673300	0.74448200
H	2.41165300	-1.03424100	1.80647400
C	4.94581000	-1.78159400	-0.34954400
H	5.48762400	-0.59483500	-2.06754800
H	4.19685700	-2.74709200	1.42675800
H	5.72212000	-2.52453600	-0.52050500
H	-3.27699800	0.20701400	2.01308300
N	-4.94537600	-0.22941400	-0.00985600
O	-5.58754000	-0.47932900	-1.03556400
O	-5.45172300	0.29449900	0.98645100
H	-1.15715500	-2.03982700	-1.87610300
F	0.28602700	-1.90655500	0.26485300

**X=F, TS3**

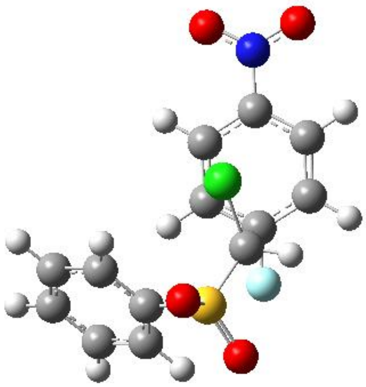

Thermochemistry:

Zero-point correction= 0.210924 (Hartree/Particle)
Thermal correction to Energy= 0.229305
Thermal correction to Enthalpy= 0.230249
Thermal correction to Gibbs Free Energy= 0.163189
Sum of electronic and zero-point Energies= -1813.926694
Sum of electronic and thermal Energies= -1813.908313
Sum of electronic and thermal Enthalpies= -1813.907369
Sum of electronic and thermal Free Energies= -1813.974430

Vibrational frequencies

**Frequency:**	**Infrared:**
-64.13	173.5335

Cartesian coordinates:

C	-1.92742000	-1.11263200	1.64582600
C	-0.83754500	-0.23555800	1.51480600
C	-0.96100600	0.95776800	0.78754800
C	-2.13220400	1.21495300	0.10608000
C	-3.20731900	0.31957800	0.19987200
C	-3.09846800	-0.84824300	0.96708000
C	0.55677400	-1.61274400	-0.11485000
S	2.27671900	-1.41538400	-0.24262800
Cl	-0.29388100	-1.40055700	-1.65948300
O	2.90167900	-1.80892200	-1.52338700
O	2.84516100	-1.95219100	1.01210400
H	-3.94710300	-1.52046600	1.02969700
H	0.36784700	-2.60904600	0.29072000
H	-0.11635500	1.63706800	0.73736600
C	2.49307900	0.36954900	-0.17376600
C	2.81459700	0.97350100	1.03954700
C	2.32632300	1.13235100	-1.32904400
C	2.96388400	2.35754400	1.09800000
H	2.93958600	0.34815100	1.91854700
C	2.46454200	2.51753500	-1.26024600
H	2.09676500	0.63681700	-2.26758200
C	2.78347800	3.13208200	-0.04903300

H	3.21824700	2.83365600	2.04261100
H	2.32749100	3.11787200	-2.15702700
H	2.89471800	4.21326400	0.00025700
H	-2.24301200	2.11182100	-0.49309600
N	-4.42890300	0.60519200	-0.48140500
O	-4.50766400	1.64637000	-1.14039400
O	-5.36260400	-0.19726000	-0.38008400
H	-1.81678400	-2.00142800	2.25898400
F	0.15605800	-0.33850400	2.41842200

X=F, TS4

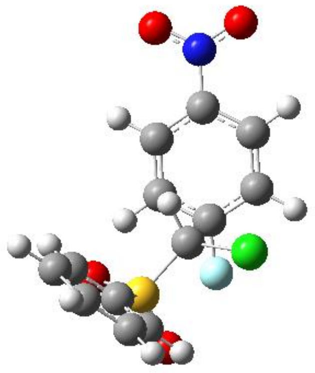

Thermochemistry:

Zero-point correction= 0.211171 (Hartree/Particle)
Thermal correction to Energy= 0.229523
Thermal correction to Enthalpy= 0.230467
Thermal correction to Gibbs Free Energy= 0.163149
Sum of electronic and zero-point Energies= -1813.931039
Sum of electronic and thermal Energies= -1813.912687
Sum of electronic and thermal Enthalpies= -1813.911743
Sum of electronic and thermal Free Energies= -1813.979061

Vibrational frequencies

**Frequency:**	**Infrared:**
-83.01	312.6812

Cartesian coordinates:

C	2.51157800	-1.46301800	0.75199200
C	1.80511600	-1.47134300	-0.46482100
C	2.11944700	-0.55193000	-1.48437600
C	3.01947700	0.45720500	-1.22652200
C	3.65904000	0.52162200	0.02371800
C	3.41015500	-0.44835000	1.00503500
C	-0.30690900	-0.49452300	0.32762200
S	-1.62208000	-0.68795800	-0.77285600
O	-2.14387800	-2.06213200	-0.72882100
O	-1.15143300	-0.11339400	-2.05093300
H	3.94055900	-0.39232700	1.94932800

H	1.58323900	-0.61395000	-2.42503200
C	-3.01724300	0.34690200	-0.28172500
C	-3.11625900	1.65161000	-0.76565000
C	-3.94571000	-0.14083500	0.63839300
C	-4.15475100	2.47070100	-0.32954500
H	-2.38723200	2.00024900	-1.49240200
C	-4.97805000	0.68685800	1.07558800
H	-3.85526100	-1.16499300	0.98800900
C	-5.08542700	1.99158800	0.59395000
H	-4.24117900	3.48475800	-0.71448400
H	-5.70509800	0.30949800	1.79172300
H	-5.89483500	2.63365600	0.93493400
H	3.24969800	1.20620600	-1.97614600
N	4.60360200	1.56022400	0.28308300
O	4.82020800	2.39523400	-0.60078400
O	5.17000800	1.58476800	1.38076500
H	2.29140400	-2.22533800	1.49176700
Cl	-0.64603700	-1.05761000	1.97697800
H	0.00334700	0.55101700	0.35169200
F	1.18266800	-2.61093000	-0.81196400

X=F, TS5

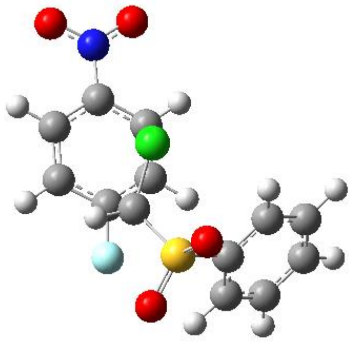

Thermochemistry:

Zero-point correction= 0.210923 (Hartree/Particle)
Thermal correction to Energy= 0.229305
Thermal correction to Enthalpy= 0.230249
Thermal correction to Gibbs Free Energy= 0.163187
Sum of electronic and zero-point Energies= -1813.926696
Sum of electronic and thermal Energies= -1813.908314
Sum of electronic and thermal Enthalpies= -1813.907370
Sum of electronic and thermal Free Energies= -1813.974432

Vibrational frequencies

**Frequency:**	**Infrared:**
-63.98	173.2746

Cartesian coordinates:

C	0.96106500	0.95746900	0.78814900

C	0.83776500	-0.23607600	1.51506700
C	1.92762600	-1.11323300	1.64551500
C	3.09857100	-0.84858600	0.96667700
C	3.20729800	0.31952300	0.19990400
C	2.13215600	1.21490600	0.10658300
C	-0.55706300	-1.61282700	-0.11488200
S	-2.27694400	-1.41520000	-0.24290900
O	-2.84567000	-1.95208400	1.01166700
O	-2.90177500	-1.80847900	-1.52381400
H	2.24284600	2.11197500	-0.49231600
H	1.81709100	-2.00226800	2.25834500
C	-2.49307000	0.36976100	-0.17385000
C	-2.32583200	1.13273800	-1.32894200
C	-2.81489100	0.97355500	1.03946100
C	-2.46385000	2.51793300	-1.25994200
H	-2.09604600	0.63733800	-2.26749600
C	-2.96398300	2.35760900	1.09811200
H	-2.94025700	0.34806700	1.91831300
C	-2.78307800	3.13232200	-0.04872700
H	-2.32641000	3.11840600	-2.15657400
H	-3.21857700	2.83359500	2.04272400
H	-2.89415400	4.21351400	0.00071700
H	3.94720600	-1.52084600	1.02889300
N	4.42878100	0.60538600	-0.48147300
O	4.50742300	1.64679400	-1.14010600
O	5.36250000	-0.19709200	-0.38057600
H	0.11638400	1.63675600	0.73829500
Cl	0.29385000	-1.40063200	-1.65938600
H	-0.36838700	-2.60918200	0.29064600
F	-0.15573500	-0.33942000	2.41875000

X=Cl, TS1

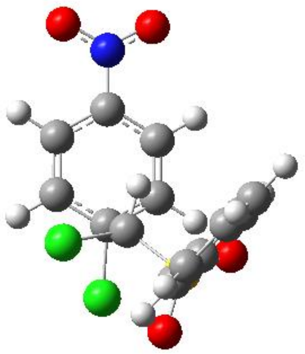

Thermochemistry:

Zero-point correction=	0.209784 (Hartree/Particle)
Thermal correction to Energy=	0.228522
Thermal correction to Enthalpy=	0.229467

Thermal correction to Gibbs Free Energy= 0.161156
Sum of electronic and zero-point Energies= -2174.211864
Sum of electronic and thermal Energies= -2174.193126
Sum of electronic and thermal Enthalpies= -2174.192181
Sum of electronic and thermal Free Energies= -2174.260492

Vibrational frequencies

**Frequency:**	**Infrared:**
-144.28	555.7168

Cartesian coordinates:

C	-2.08994800	-0.60071800	-1.35254200
C	-1.79268100	-1.38363200	-0.20851500
C	-2.47657000	-1.11997900	1.00349800
C	-3.25635800	0.00953000	1.12148600
C	-3.44560500	0.84847600	0.01349300
C	-2.87320800	0.52221100	-1.22972300
C	0.31578900	-0.46169100	0.38421300
S	1.64171000	-0.71246500	-0.70600700
Cl	0.67854200	-0.87614700	2.06532700
O	1.14594300	-0.29062300	-2.03082000
O	2.21960100	-2.05109200	-0.52221100
H	-3.06807000	1.16106700	-2.08415100
H	-0.02291500	0.57306200	0.31905700
H	-2.33019800	-1.78423500	1.84829900
C	2.97860100	0.43166900	-0.31298800
C	3.93444600	0.07185300	0.63730900
C	3.00585300	1.69327200	-0.90760800
C	4.92440400	0.98570200	0.99292400
H	3.89803700	-0.92173200	1.07419700
C	4.00168600	2.59893600	-0.55078200
H	2.25631100	1.94126300	-1.65443000
C	4.96040000	2.24845300	0.40132600
H	5.67336100	0.70880100	1.73181700
H	4.03282800	3.57992000	-1.02019300
H	5.73618800	2.95855900	0.67947500
H	-3.74811700	0.25251600	2.05728300
N	-4.27009400	2.00301300	0.13351600
O	-4.78566500	2.25304900	1.22978200
O	-4.43984800	2.71500300	-0.86318300
H	-1.62376400	-0.85412800	-2.29811400
Cl	-1.23274500	-3.02455500	-0.46255300

X=Cl, TS2

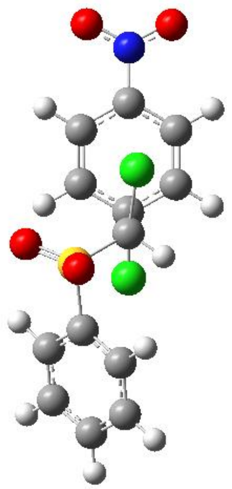

Thermochemistry:

Zero-point correction= 0.209300 (Hartree/Particle)
Thermal correction to Energy= 0.228175
Thermal correction to Enthalpy= 0.229119
Thermal correction to Gibbs Free Energy= 0.160630
Sum of electronic and zero-point Energies= -2174.200624
Sum of electronic and thermal Energies= -2174.181749
Sum of electronic and thermal Enthalpies= -2174.180805
Sum of electronic and thermal Free Energies= -2174.249294

Vibrational frequencies

**Frequency:**	**Infrared:**
-142.84	364.3805

Cartesian coordinates:

C	-1.77989300	-1.25110000	-1.29920000
C	-0.90518900	-1.20687600	-0.19250700
C	-1.41066600	-0.84636400	1.07589500
C	-2.72228300	-0.44914700	1.20586900
C	-3.57918900	-0.46943600	0.09283600
C	-3.09506900	-0.85820300	-1.16146300
C	0.38382200	0.83770900	-0.72022100
S	1.58469200	1.49230100	0.38582400
Cl	-0.94227900	2.00140700	-0.91687400
O	1.11550100	1.35150600	1.77867900
O	2.15443400	2.80581000	0.00762300
H	-3.77195100	-0.87142800	-2.00884300
H	0.86963800	0.68794400	-1.69075300
H	-0.74044300	-0.82428000	1.92813100
C	2.92838400	0.31173100	0.16758700
C	3.73980200	0.39270500	-0.96201600
C	3.17983300	-0.62590900	1.16315400
C	4.80055200	-0.49837300	-1.10760000
H	3.54883800	1.16173300	-1.70588700
C	4.25085000	-1.50539300	1.01956100

H	2.53377600	-0.64996500	2.03578800
C	5.05565900	-1.44787500	-0.11763600
H	5.43533900	-0.44468000	-1.98926700
H	4.45356400	-2.24153500	1.79418100
H	5.88776800	-2.13954200	-0.23071000
H	-3.11197500	-0.13379600	2.16742900
N	-4.94793400	-0.10750600	0.24099700
O	-5.67877000	-0.13107200	-0.75696600
O	-5.35743300	0.21095400	1.36278500
H	-1.40293600	-1.58343800	-2.26141800
Cl	0.50871000	-2.25338900	-0.26085100

X=Cl, TS3

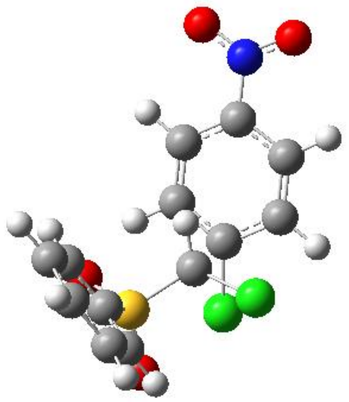

Thermochemistry:

Zero-point correction= 0.209784 (Hartree/Particle)
Thermal correction to Energy= 0.228522
Thermal correction to Enthalpy= 0.229467
Thermal correction to Gibbs Free Energy= 0.161156
Sum of electronic and zero-point Energies= -2174.211864
Sum of electronic and thermal Energies= -2174.193125
Sum of electronic and thermal Enthalpies= -2174.192181
Sum of electronic and thermal Free Energies= -2174.260491

Vibrational frequencies

**Frequency:**	**Infrared:**
-144.33	555.8805

Cartesian coordinates:

C	2.47659700	-1.11999100	1.00349200
C	1.79270200	-1.38368600	-0.20851300
C	2.08985200	-0.60070300	-1.35252700
C	2.87297600	0.52231700	-1.22969000
C	3.44536400	0.84861600	0.01352500
C	3.25625000	0.00961000	1.12149400
C	-0.31575900	-0.46193100	0.38423600
S	-1.64165600	-0.71249900	-0.70607800

O	-2.21966900	-2.05109300	-0.52243200
O	-1.14580000	-0.29056300	-2.03082600
H	3.74800400	0.25262700	2.05728600
H	1.62366000	-0.85413000	-2.29809100
C	-2.97845600	0.43171800	-0.31299500
C	-3.00554200	1.69340100	-0.90745300
C	-3.93439700	0.07187300	0.63719400
C	-4.00130600	2.59911800	-0.55057200
H	-2.25592900	1.94141500	-1.65419600
C	-4.92428600	0.98577400	0.99286300
H	-3.89811100	-0.92177300	1.07395400
C	-4.96011700	2.24860600	0.40142800
H	-4.03231900	3.58016700	-1.01985600
H	-5.67332000	0.70885100	1.73167200
H	-5.73585200	2.95875300	0.67962000
H	3.06772900	1.16122800	-2.08410100
N	4.26970500	2.00325300	0.13356700
O	4.43933200	2.71530800	-0.86310900
O	4.78528200	2.25331300	1.22982600
H	2.33033000	-1.78429300	1.84827500
Cl	-0.67858800	-0.87662600	2.06526900
H	0.02298500	0.57281900	0.31923600
Cl	1.23299700	-3.02469800	-0.46256800

X=Cl, TS4

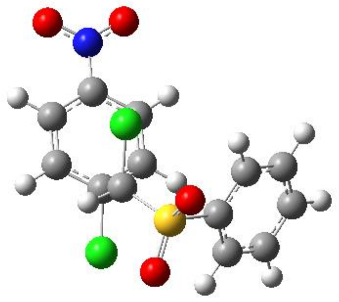

Thermochemistry:

Zero-point correction= 0.209602 (Hartree/Particle)
Thermal correction to Energy= 0.228321
Thermal correction to Enthalpy= 0.229265
Thermal correction to Gibbs Free Energy= 0.161501
Sum of electronic and zero-point Energies= -2174.207310
Sum of electronic and thermal Energies= -2174.188591
Sum of electronic and thermal Enthalpies= -2174.187647
Sum of electronic and thermal Free Energies= -2174.255410

Vibrational frequencies

**Frequency:**	**Infrared:**
-140.03	351.3833

Cartesian coordinates:			
C	0.90090000	0.62490000	1.02044500
C	0.78601200	-0.76151900	1.27308200
C	1.90848600	-1.59370400	1.04785900
C	3.05154000	-1.08190400	0.47414800
C	3.13111900	0.28424800	0.17002900
C	2.04292200	1.12936800	0.43935000
C	-0.60091800	-1.52996200	-0.57777800
S	-2.29213200	-1.13285800	-0.76173400
O	-3.01195800	-1.89285400	0.27955400
O	-2.81021300	-1.15657600	-2.14415000
H	2.12664000	2.18619700	0.21038900
H	1.84883600	-2.64484200	1.31302900
C	-2.36896500	0.59495200	-0.27614500
C	-1.98639400	1.58651400	-1.17913400
C	-2.80624900	0.92361300	1.00454800
C	-2.02595400	2.92168700	-0.78303100
H	-1.66673600	1.30803500	-2.17870800
C	-2.85438300	2.26233600	1.38876700
H	-3.09945800	0.12474700	1.67880500
C	-2.45929500	3.26164600	0.49889900
H	-1.71972000	3.69909600	-1.47933200
H	-3.19724600	2.52515300	2.38711800
H	-2.49105300	4.30566800	0.80308800
H	3.90761500	-1.71769600	0.27572600
N	4.32387700	0.81767300	-0.39003000
O	4.37169100	2.02839300	-0.63927400
O	5.27238900	0.05251800	-0.60438600
H	0.06861700	1.28005800	1.25633100
Cl	0.37144000	-1.06884900	-1.98020600
H	-0.53072200	-2.60348400	-0.39040800
Cl	-0.35544000	-1.28678800	2.50383700

Transition state structures of the reaction between 2-deutero,4-bromonitrobenzene and carbanion of chloromethyl phenyl sulfone ($PhSO_2CHCl^-$)

4-Br-2-D, TS1

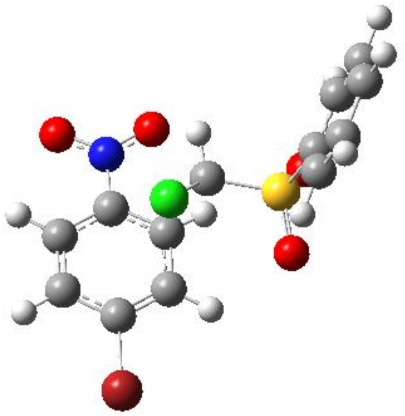

Thermochemistry:

Zero-point correction= 0.206067 (Hartree/Particle)
Thermal correction to Energy= 0.225216
Thermal correction to Enthalpy= 0.226160
Thermal correction to Gibbs Free Energy= 0.156190
Sum of electronic and zero-point Energies= -4287.973379
Sum of electronic and thermal Energies= -4287.954230
Sum of electronic and thermal Enthalpies= -4287.953286
Sum of electronic and thermal Free Energies= -4288.023257

Vibrational frequencies

**Frequency:**	**Infrared:**
-96.80	249.1084

Cartesian coordinates:

C	-2.06211000	1.97726200	0.07193100
C	-1.37044800	1.08911600	-0.78033800
C	-1.88471600	-0.22795800	-0.88345300
C	-2.90857200	-0.62409100	-0.06025500
C	-3.52831400	0.24361700	0.86326900
C	-3.09569000	1.54945700	0.91857500
C	0.72784200	0.62887600	0.25506800
S	1.76020800	-0.06634500	-0.93728900
N	-1.68247900	3.35055000	0.09694500
O	-2.31159300	4.13112100	0.82530100
Cl	0.41809800	-0.39789200	1.65561700
O	1.92835000	0.97103700	-1.97324000
O	1.26092300	-1.40087800	-1.31322900
O	-0.73596600	3.70877800	-0.60785500
H	-0.72488800	1.48917900	-1.55055300
H	-3.54968100	2.26659700	1.59371300
H	-4.32445200	-0.11485100	1.50666000
H	1.08176200	1.61511600	0.55198900
H	-1.41056900	-0.92881000	-1.56201000
C	3.40759500	-0.35863300	-0.27142200
C	3.67672900	-1.54708700	0.40807600

C	4.37711000	0.63976600	-0.37180300
C	4.92985400	-1.73472600	0.98793900
H	2.90968500	-2.31402700	0.45848900
C	5.62820600	0.44197800	0.20635000
H	4.14227300	1.55003600	-0.91711300
C	5.90615700	-0.74338800	0.88947700
H	5.14587100	-2.66118300	1.51581500
H	6.39106900	1.21289000	0.11967200
H	6.88415300	-0.89516600	1.34122600
Br	-3.54269800	-2.40381500	-0.16381900

12
12
12
12
12
12
12
32
14
16
35
16
16
16
1
2
1
1
1
12
12
12
12
1
12
1
12
1
1
1
79

4-Br-2-D, TS2

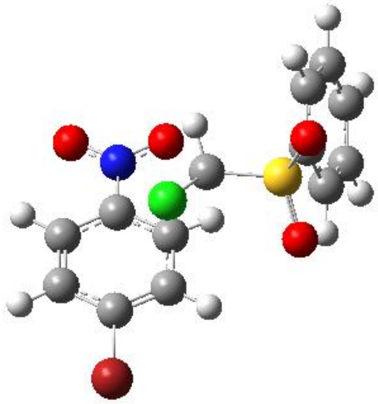

Thermochemistry:

Zero-point correction= 0.205804 (Hartree/Particle)
Thermal correction to Energy= 0.225030
Thermal correction to Enthalpy= 0.225974
Thermal correction to Gibbs Free Energy= 0.155873
Sum of electronic and zero-point Energies= -4287.964262
Sum of electronic and thermal Energies= -4287.945036
Sum of electronic and thermal Enthalpies= -4287.944092
Sum of electronic and thermal Free Energies= -4288.014193

Vibrational frequencies

**Frequency:**	**Infrared:**
-41.50	55.2438

Cartesian coordinates:

C	0.76002600	2.13461800	-0.21652900
C	0.42989800	0.95189300	0.46787800
C	1.48193600	0.08917300	0.83161300
C	2.75756700	0.36169500	0.39410200
C	3.07422500	1.50746200	-0.35959700
C	2.06260600	2.39363400	-0.66342300
C	-0.61862600	-0.68244600	-1.18152100
S	-1.63023900	-1.87782300	-0.41129700
N	-0.26616400	3.09083700	-0.48826100
O	0.03668000	4.13346500	-1.08303100
Cl	0.80290000	-1.43141500	-1.93875300
O	-0.90267500	-2.47485400	0.72940200
O	-2.35904400	-2.81721500	-1.29596900
O	-1.41421500	2.83625700	-0.12048700
H	-0.55148000	0.84891800	0.90512300
H	2.25507500	3.29795800	-1.23019700
H	4.09131100	1.68071600	-0.69448000
H	-1.20384600	-0.15877500	-1.94215900
H	1.25794200	-0.82134500	1.37721500
C	-2.88215500	-0.78518400	0.27930600
C	-3.90634500	-0.30564200	-0.53436200
C	-2.82732900	-0.45321100	1.63018000

C	-4.86978100	0.54063500	0.00802500
H	-3.94882700	-0.60745500	-1.57734500
C	-3.80220800	0.38339100	2.16949200
H	-2.02947200	-0.86732900	2.24102100
C	-4.81797800	0.88646300	1.35812400
H	-5.66473400	0.92990200	-0.62390100
H	-3.76391600	0.64880600	3.22361600
H	-5.57064500	1.55014900	1.77778700
Br	4.15683700	-0.83755100	0.81604800

12
12
12
12
12
12
12
32
14
16
35
16
16
16
1
2
1
1
1
12
12
12
12
1
12
1
12
1
1
1
79

4-Br-2-D, TS3

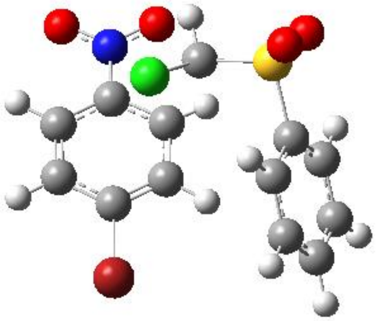

Thermochemistry:

Zero-point correction= 0.206017 (Hartree/Particle)
Thermal correction to Energy= 0.225106
Thermal correction to Enthalpy= 0.226050
Thermal correction to Gibbs Free Energy= 0.156667
Sum of electronic and zero-point Energies= -4287.967686
Sum of electronic and thermal Energies= -4287.948598
Sum of electronic and thermal Enthalpies= -4287.947654
Sum of electronic and thermal Free Energies= -4288.017037

Vibrational frequencies

**Frequency:**	**Infrared:**
-90.31	176.2104

Cartesian coordinates:

C	2.24802600	-1.32158600	-0.56697800
C	0.98575900	-0.79195000	-0.91265500
C	0.80871200	0.59857500	-0.71787200
C	1.76383900	1.32316700	-0.04705200
C	2.97623800	0.75906400	0.39412800
C	3.21153900	-0.57086200	0.11982900
C	-0.51128600	-1.73964200	0.74727100
S	-2.23011500	-1.69370000	0.48739600
N	2.55350300	-2.67094700	-0.91676100
O	3.66817700	-3.12444900	-0.62569700
Cl	0.00063800	-0.95920800	2.25273700
O	-3.07590900	-1.71073900	1.69751900
O	-2.50656200	-2.66332500	-0.59239900
O	1.69047600	-3.33195300	-1.49849500
H	0.35022700	-1.33827300	-1.59534200
H	4.14004400	-1.05079400	0.40875700
H	3.70394800	1.36097700	0.92751900
H	-0.18745300	-2.78041700	0.71108000
H	-0.09961000	1.07803800	-1.06411500
C	-2.49323800	-0.06620600	-0.22937300
C	-2.65189000	0.04896900	-1.60890500
C	-2.50344700	1.06350700	0.58875600
C	-2.81168400	1.31126800	-2.17773500
H	-2.64907700	-0.85423600	-2.21301700

C	-2.64868800	2.32250900	0.01046700
H	-2.39235600	0.95091300	1.66296800
C	-2.80196000	2.44931700	-1.37045800
H	-2.93691300	1.40734800	-3.25414400
H	-2.63556200	3.20898000	0.64014700
H	-2.91198100	3.43498000	-1.81713800
Br	1.47926400	3.17176500	0.24833600

12
12
12
12
12
12
12
32
14
16
35
16
16
16
1
2
1
1
1
12
12
12
12
1
12
1
12
1
1
1
79

4-Br-2-D, TS4

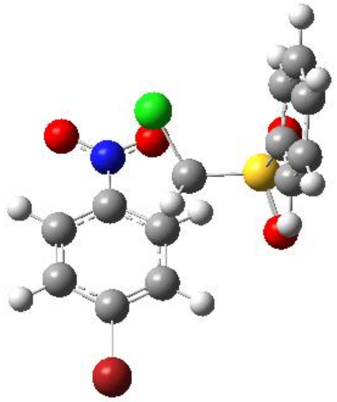

Thermochemistry:

Zero-point correction= 0.206084 (Hartree/Particle)
Thermal correction to Energy= 0.225244
Thermal correction to Enthalpy= 0.226188
Thermal correction to Gibbs Free Energy= 0.156110
Sum of electronic and zero-point Energies= -4287.971853
Sum of electronic and thermal Energies= -4287.952693
Sum of electronic and thermal Enthalpies= -4287.951749
Sum of electronic and thermal Free Energies= -4288.021827

Vibrational frequencies

**Frequency:**	**Infrared:**
-68.85	185.8557

Cartesian coordinates:

C	2.16837900	1.75766300	-0.00872300
C	1.52323900	0.86891700	0.87213600
C	1.97755500	-0.47285700	0.88956500
C	2.84947800	-0.89828400	-0.08126600
C	3.36551100	-0.03646700	-1.07068100
C	3.02704500	1.30012900	-1.01607900
C	-0.60507400	0.44633000	-0.17195300
S	-1.79549600	0.08314200	1.01895400
N	1.94332300	3.16647800	0.12129900
O	2.46313100	3.92387700	-0.70674400
O	-2.18166800	1.29799100	1.75100000
O	-1.26146300	-1.08326200	1.75463300
O	1.27128100	3.56591700	1.07008200
H	0.95334500	1.26565200	1.70104600
H	3.43551000	2.01410600	-1.72283800
H	4.03691500	-0.41491200	-1.83407900
H	1.56127900	-1.16192100	1.61657800
C	-3.31573900	-0.49931000	0.24592100
C	-3.49813200	-1.86372600	0.01862300
C	-4.26501600	0.42670700	-0.18852000
C	-4.64246800	-2.30337700	-0.64199800

H	-2.74912600	-2.56264700	0.38129700
C	-5.40334100	-0.02076000	-0.85589100
H	-4.10778600	1.48195700	0.01424600
C	-5.59530700	-1.38363500	-1.08344900
H	-4.79436000	-3.36784900	-0.80875800
H	-6.14652000	0.69802700	-1.19447200
H	-6.48756300	-1.72949000	-1.60097200
Cl	-0.98853600	1.77215600	-1.26636600
H	-0.34400600	-0.45228300	-0.73040300
Br	3.39214500	-2.71305900	-0.10981900

12
12
12
12
12
12
12
32
14
16
16
16
16
1
2
1
1
12
12
12
12
1
12
1
12
1
1
1
35
1
79

S8. References.

(1) Frisch, M. J.; Trucks, G. W.; Schlegel, H. B.; Scuseria, G. E.; Robb, M. A.; Cheeseman, J. R.; Scalmani, G.; Barone, V.; Mennucci, B.; Petersson, G. A.; Nakatsuji, H.; Caricato, M.; Li, X.; Hratchian, H. P.; Izmaylov, A. F.; Bloino, J.; Zheng, G.; Sonnenberg, J. L.; Hada, M.; Ehara, M.; Toyota, K.; Fukuda, R.; Hasegawa, J.; Ishida, M.; Nakajima, T.; Honda, Y.; Kitao, O.; Nakai, H.; Vreven, T.; Montgomery Jr., J. A.; Peralta, J. E.; Ogliaro, F.; Bearpark, M. J.; Heyd, J.; Brothers, E. N.; Kudin, K. N.; Staroverov, V. N.; Kobayashi, R.; Normand, J.; Raghavachari, K.; Rendell, A. P.; Burant, J. C.; Iyengar, S. S.; Tomasi, J.; Cossi, M.; Rega, N.; Millam, N. J.; Klene, M.; Knox, J. E.; Cross, J. B.; Bakken, V.; Adamo, C.; Jaramillo, J.; Gomperts, R.; Stratmann, R. E.; Yazyev, O.; Austin, A. J.; Cammi, R.; Pomelli, C.; Ochterski, J. W.; Martin, R. L.; Morokuma, K.; Zakrzewski, V. G.; Voth, G. A.; Salvador, P.; Dannenberg, J. J.; Dapprich, S.; Daniels, A. D.; Farkas, Ö.; Foresman, J. B.; Ortiz, J. V.; Cioslowski, J.; Fox, D. J.; Gaussian, Inc.: Wallingford, CT, USA, 2009.

(2) Adamo, C.; Barone, V. *J. Chem. Phys.* **1999**, *110*, 6158.

(3) Kruszewski, J.; Krygowski, T. M. *Tetrahedron Lett.* **1972**, *13*, 3839.

(4) Parr, R. G.; Yang, W. *J. Am. Chem. Soc.* **1984**, *106*, 4049.

(5) Ormazábal-Toledo, R.; Contreras, R.; Campodónico, P. R. *J. Org. Chem.* **2013**, *78*, 1091.

(6) Ormazabal-Toledo, R.; Contreras, R.; Tapia, R. A.; Campodonico, P. R. *Org. Biomol. Chem.* **2013**, *11*, 2302.

(7) Błażej, S.; Mąkosza, M. *Chem. -- Eur. J.* **2008**, *14*, 11113.

(8) Błaziak, K.; Danikiewicz, W.; Mąkosza, M. *J. Am. Chem. Soc.* **2016**, *138*, 7276.

(9) Hirshfeld, F. L. *Theor. Chim. Acta* **1977**, *44*, 129.

(10) Jaramillo, P.; Pérez, P.; Contreras, R.; Tiznado, W.; Fuentealba, P. *J. Phys. Chem. A* **2006**, *110*, 8181.

(11) Parr, R. G.; Szentpály, L. v.; Liu, S. *J. Am. Chem. Soc.* **1999**, *121*, 1922.

(12) Parr, R. G. *Density-Functional Theory of Atoms and Molecules*; Oxford University Press: New York, USA, 1989.

(13) Pearson, R. *J. Chem. Sci. (Bangalore, India)* **2005**, *117*, 369.

(14) Eyring, H. *J. Chem. Phys.* **1935**, *3*, 107.

(15) Evans, M. G.; Polanyi, M. *Transactions of the Faraday Society* **1935**, *31*, 875.

(16) Mąkosza, M.; Lemek, T.; Kwast, A.; Terrier, F. *J. Org. Chem.* **2002**, *67*, 394.
